# Modified mRNA/lipid nanoparticle-based vaccines expressing respiratory syncytial virus F protein variants are immunogenic and protective in rodent models of RSV infection

**DOI:** 10.1038/s41541-020-0163-z

**Published:** 2020-02-14

**Authors:** Amy S. Espeseth, Pedro J. Cejas, Michael P. Citron, Dai Wang, Daniel J. DiStefano, Cheryl Callahan, Gregory O’ Donnell, Jennifer D. Galli, Ryan Swoyer, Sinoeun Touch, Zhiyun Wen, Joseph Antonello, Lan Zhang, Jessica A. Flynn, Kara S. Cox, Daniel C. Freed, Kalpit A. Vora, Kapil Bahl, Andrew H. Latham, Jeffrey S. Smith, Marian E. Gindy, Giuseppe Ciaramella, Daria Hazuda, Christine A. Shaw, Andrew J. Bett

**Affiliations:** 1 ID/Vaccines Discovery, Merck & Co., Inc., Kenilworth, NJ USA; 2 Pharmacology, Merck & Co., Inc., Kenilworth, NJ USA; 3 Biostatistics, Merck & Co., Inc., Kenilworth, NJ USA; 4 Moderna, Inc., Cambridge, MA USA; 5 Pharmaceutical Science, Merck & Co., Inc., Kenilworth, NJ USA

**Keywords:** Vaccines, Virology, RNA vaccines

## Abstract

The RSV Fusion (F) protein is a target for neutralizing antibody responses and is a focus for vaccine discovery; however, the process of RSV entry requires F to adopt a metastable prefusion form and transition to a more stable postfusion form, which displays less potent neutralizing epitopes. mRNA vaccines encode antigens that are translated by host cells following vaccination, which may allow conformational transitions similar to those observed during natural infection to occur. Here we evaluate a panel of chemically modified mRNA vaccines expressing different forms of the RSV F protein, including secreted, membrane associated, prefusion-stabilized, and non-stabilized structures, for conformation, immunogenicity, protection, and safety in rodent models. Vaccination with mRNA encoding native RSV F elicited antibody responses to both prefusion- and postfusion-specific epitopes, suggesting that this antigen may adopt both conformations in vivo. Incorporating prefusion stabilizing mutations further shifts the immune response toward prefusion-specific epitopes, but does not impact neutralizing antibody titer. mRNA vaccine candidates expressing either prefusion stabilized or native forms of RSV F protein elicit robust neutralizing antibody responses in both mice and cotton rats, similar to levels observed with a comparable dose of adjuvanted prefusion stabilized RSV F protein. In contrast to the protein subunit vaccine, mRNA-based vaccines elicited robust CD4+ and CD8+ T-cell responses in mice, highlighting a potential advantage of the technology for vaccines requiring a cellular immune response for efficacy.

## Introduction

Respiratory syncytial virus (RSV) is a member of the *Pneumoviridae* family that causes upper and lower respiratory tract illness worldwide, with substantial morbidity in infants, the immune compromised, and older adults. Worldwide, ~1.5% of infants are hospitalized with RSV lower respiratory tract infection (LRTI) with an estimated mortality rate of 118,200–149,400 deaths in children under the age of 5 every year.^1^ In adults, the severity of medically attended RSV infection increases with age. On average, ~5% of older adults are infected with RSV annually, resulting in an estimated 177,000 hospitalizations and 14,000 deaths each year.^2^ Despite the clear medical need for an RSV vaccine and the advancement of multiple candidates to clinical trials, a licensed RSV vaccine is not yet available.

The RSV F protein is a type I fusion glycoprotein that is conserved between clinical isolates, including the RSV-A and RSV-B antigenic subgroups. RSV F protein is an attractive vaccine target, both because it is relatively well conserved among serotypes and because neutralizing antibodies elicited by natural RSV infection predominantly target RSV F.^3^ F protein transitions between two well-characterized conformations; a metastable prefusion conformation and a stable postfusion conformation. Although epitopes targeted by neutralizing monoclonal antibodies exist on both conformations, characterization of the natural human immune response to RSV infection revealed that most RSV-neutralizing antibodies bind the prefusion conformation of the F protein.^4,5^ The elucidation of the crystal structure of RSV F protein trapped in the prefusion conformation through binding to a prefusion-specific monoclonal antibody has facilitated sophisticated structure-based design predictions of mutations that stabilize the prefusion conformation and generated improved immune responses to RSV in preclinical models.^6–13^ One of these stabilized forms of RSV, termed DS-Cav1, has demonstrated robust immunogenicity in a Phase 1 clinical trial.^14^

Developing an RSV vaccine poses distinct sets of challenges for both the infant and older adult target populations. Protection provided by passive transfer of maternal antibodies or palivizumab, a monoclonal antibody targeting RSV F, in RSV-naïve infants suggests that neutralizing antibodies are sufficient to protect against severe LRTI caused by RSV in this population.^15^ However, the development of whole inactivated or subunit-based vaccines for infants has been stymied by the immune pathology elicited by a formalin-inactivated RSV vaccine candidate (FI-RSV).^16,17^ In contrast to infants, the vast majority of adults have been naturally infected with RSV and have detectable neutralizing antibody titers. While the immune requirements for protection against RSV-associated disease in the elderly are less understood, lower humoral responses to RSV F and G proteins, and a decrease in nasal RSV-specific IgA have been identified as risk factors for RSV disease.^18–22^ Waning cellular immunity may also play a role in RSV infection in the elderly, as older adults have fewer RSV-specific CD8+ T-cells and increased numbers of regulatory T-cells, with a bias towards a Th2 functional phenotype.^23–27^

Nucleic acid-based vaccines consisting of in vitro transcribed mRNAs encapsulated within lipid nanoparticles (LNPs) for effective cellular delivery have the potential to transform vaccine research and development. They allow for the rapid generation of candidate antigens for preclinical evaluation, and elicit strong humoral and cellular immune responses, as well as offering a consistent platform for vaccine manufacturing. Formulation of the mRNA antigen within an LNP improves the immune response to the vaccine, presumably by both protecting the mRNA from enzymatic degradation and facilitating efficient uptake and intracellular release of the mRNA in target cells.^28^ For effective mRNA immunization, the vaccine mRNA may be modified to incorporate naturally occurring bases to reduce the inflammatory response to the vaccine.^29–33^ Importantly, immunization with chemically modified mRNA encapsulated within a lipid nanoparticle was found to be safe and immunogenic in humans following a Phase 1 clinical trial using an mRNA vaccine targeting H10N8 influenza.^34,35^

Here we evaluated LNP-encapsulated chemically modified mRNA vaccines encoding various forms of RSV F protein in rodent models of immunogenicity and protection against RSV challenge. We discovered that mRNA/LNP vaccine candidates expressing either prefusion stabilized or native forms of RSV F protein elicit robust neutralizing antibody responses in both mouse and cotton rat, similar to levels observed with a comparable dose of adjuvanted prefusion stabilized RSV F protein (DS-Cav1).^7^ In contrast to the protein-based vaccine, mRNA/LNP vaccines elicit robust cellular immune responses to RSV F. Additionally, cotton rats vaccinated with mRNA/LNP vaccines are completely protected against challenge with RSV-A and RSV-B and did not develop vaccine enhanced respiratory disease (VERD), unlike cotton rats immunized with FI-RSV. Together the data suggest that mRNA/LNP vaccines expressing forms of RSV F protein have the potential to be safe and effective at prevention of RSV disease.

## Results

### mRNA antigen design

**Fig. 1:**
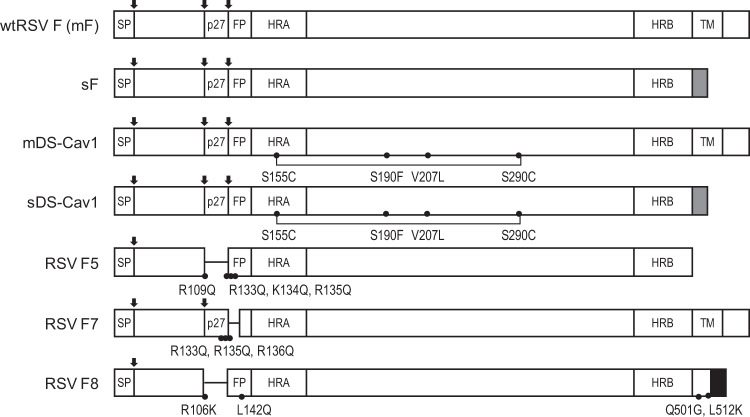
Variants of RSV F protein expressed by mRNA vaccines. The different forms of RSV protein expressed by the modified mRNA vaccines evaluated in this study are shown. wtRSV (mF) expresses the full length RSV F protein from the RSV A2 strain. The locations of the signal peptide (SP), p27 peptide (p27), fusion peptide (FP), heptad repeat A (HRA), heptad repeat B (HRB), and transmembrane peptide (TM) are shown. For each construct, arrows indicated proteolytic cleavage sites that remain in the expressed protein. Deleted amino acid sequences are indicated with a horizontal line replacing the bar. Point mutations added to stabilize the prefusion conformation of the antigen are indicated with a solid circle (•) and the identity of the amino acid change is indicated below the circle. The newly generated disulfide bonds formed in the DS-Cav1-based constructs is indicated with a bracket. Secreted constructs in which the transmembrane domain and cytoplasmic sequence are deleted and replaced with a foldon or GCN4 peptide sequence to support trimerization are indicated with a gray or black-filled bar, respectively.

The RSV F surface protein elicits the majority of neutralizing antibodies following natural infection in humans, and as a result it is the antigen targeted by the majority of RSV vaccine candidates.^3^ Since RSV F is challenging to express and purify, many publications have described forms of the antigen designed to improve its expression in different cell substrates or to stabilize the desired conformation of the antigen during purification. Vaccination using mRNA allows the opportunity to efficiently explore multiple antigen designs. We thus designed a panel of mRNA antigens to compare the immunogenicity of different forms of the RSV F protein (Table 1 and Fig. 1). Our antigens were designed to explore the impact of expressing secreted versus membrane-associated, prefusion stabilized versus native, and trimeric versus monomeric forms of F protein. To that end, we included the following RSV F protein variants (Table 1 and Fig. 1): full length wild type RSV F (mF), a truncated secreted trimeric form of RSV F (sF), the secreted prefusion stabilized DS-Cav1 construct described in McLellan et al.^7^ (sDS-Cav1), a full length RSV F, including the four point mutations present in DS-Cav1 (mDS-Cav1), a monomeric prefusion single chain form of RSV F as described in Swanson et al.^8^ (RSV F5), a form of full length RSV F as described in Smith et al.^13^ (RSV F7), and a prefusion stabilized form of RSV F as described in Blais et al.^11^ (RSV F8).

**Table 1:** mRNA constructs expressing forms of RSV F protein designed for evaluation of immunogenicity in BALB/c mice.

	Signal peptide	Transmembrane or Secreted	Trimerization Domain	Prefusion stabilized	Reference
wtRSV F (mF)	Native	Transmembrane	Membrane	No	
sF	Native	Secreted	Foldon^a^	No	
mDS-Cav1	Native	Transmembrane	Membrane	Yes	^7^
sDS-Cav1	Native	Secreted	Foldon	Yes	^7^
RSV F5	Native	Secreted	Foldon	Yes	^8^
RSV F7	Native	Transmembrane	Membrane	No	^13^
RSV F8	Native	Secreted	GCN4^b^	Yes	^11^

^a^T4 phage fibritin trimerization domain (Foldon).^58^

^b^*S. cerevisiae* GCN4 trimerization domain.^59^

### In vitro characterization of expressed antigens from mRNA vaccines

In vitro characterization of the expressed protein was conducted following transfection of the mRNA constructs into cells. The conformation of transmembrane forms of RSV F were evaluated using flow cytometry. Increasing amounts of mRNAs expressing transmembrane forms of RSV F (mF, mDS-Cav1, RSV F7) were transfected into Expi293F cells. The transfected cells were stained with conformation-indicating antibodies, including D25, 4D7, and AM14, and analyzed by flow cytometry. Binding of D25 antibody indicates the presence of the antigenic site Φ found on prefusion F protein, binding of 4D7 indicates postfusion RSV F or an intermediate conformation, and binding of AM14 indicates the trimeric form of prefusion RSV F.^6,36,37^ The results are summarized in Table 2 and shown in Supplementary Fig. 1. The mF mRNA led to expression of a mixed population of prefusion and postfusion RSV F. Of the cells expressing RSV F, close to half bound only to 4D7, with another 30–40% binding both 4D7 and D25 or 4D7 and AM14. These observations suggest that, as has been described for RSV F in the context of the virus, the RSV F protein is initially expressed on the surface of the cell in its trimeric prefusion conformation, but then can flip into the more thermodynamically stable postfusion conformation. Similar to mF mRNA, cells transfected with RSV F7 mRNA primarily bound 4D7. Approximately two-thirds of the cells expressing RSV F7 bound to both 4D7 and D25; however, the lack of AM14 binding suggested that the protein did not complete maturation into its fully cleaved and trimeric conformation. In contrast, the mDS-Cav1 mRNA transfected cells had ~3-fold higher D25 binding relative to 4D7 binding and roughly twice as many cells binding AM14 as bound 4D7, indicating the mRNA could be translated into trimeric prefusion F protein in vitro.

**Table 2:** Percentage of cells transfected with mRNAs expressing membrane-associated forms of RSV F protein that are bound by D25, 4D7, or AM14.

mRNA (µg)	D25+ (%)	4D7+ (%)	AM14+ (%)
*mF*			
0.17	2.2	1.3	3.0
0.3	6.0	2.1	7.9
0.8	20.4	13.7	9.4
1.7	27.4	39.7	20.8
2.5	26.1	40.4	22.0
*mDS-Cav1*			
0.17	0.4	0.6	0.4
0.3	2.7	1.7	1.9
0.8	15.4	3.9	8.8
1.7	36.7	14.9	21.6
2.5	44.9	12.4	27.4
*RSV F7*			
0.17	4.9	18.5	0.0
0.3	10.3	32.9	0.0
0.8	45.7	80.6	0.7
1.7	63.2	88.9	0.8
2.5	62.7	91.7	0.5
*no mRNA*			
–	0.0	0.0	0.0
–	0.0	0.0	0.0
–	0.0	0.0	0.2
–	0.1	0.3	1.4
–	0.6	0.8	0.5

The conformation of secreted forms of RSV F (sF, sDS-Cav1, RSV F5, RSV F8) was evaluated by sandwich ELISA of culture medium taken from cells transfected with their respective mRNAs (Table 3). As expected, the sDS-Cav1 and RSV F8 secreted proteins both bound to D25, confirming the stabilization of these proteins in the prefusion conformation. The sDS-Cav1 protein was the only secreted protein to bind AM14, indicating that this form of F retains its prefusion and trimeric conformation following secretion, while the RSV F8 protein appeared to have a mixture of prefusion and postfusion conformations with binding to both D25 and 4D7. Unlike the mF protein, the sF protein bound only to 4D7 and did not exhibit any evidence of binding to prefusion-specific antibodies.

**Table 3:** Binding of secreted forms of RSV F protein to conformation-indicating antibodies following transfection of modified mRNAs into Expi293F cells.

	D25 binding (ng/mL)	4D7 binding (ng/mL)	AM14 binding (ng/mL)
sF	0.0	28.3	0.0
sDS-Cav1	30.0	2.8	25.7
RSV F5	1.8	2.0	0.0
RSV F8	14.7	30.0	0.0
No mRNA	0.0	0.0	0.0

### mRNA/LNP vaccines elicit humoral responses in mice

BALB/c mice were immunized with the different mRNAs described in Table 1 in two intramuscular immunizations of 10 μg mRNA 3 weeks apart. Immunization with 10 μg of the DS-Cav1 protein antigen formulated with Adju-Phos®, an aluminum phosphate-based adjuvant, was included as a control. Sera were collected 2 weeks following the second immunization and tested for binding to prefusion and postfusion forms of RSV F protein by ELISA, and for neutralization of RSV-A (Long). mRNA/LNP vaccines were found to be highly immunogenic as measured by high levels of serum antibody binding RSV F protein, and by serum neutralizing antibody titers (Table 4). For most of the mRNA antigens evaluated, the serological immune responses were comparable with or greater than vaccinations using aluminum adjuvanted DS-Cav1 protein.

**Table 4:** Serological response to RSV in BALB/c mice following two immunizations with 10 μg of selected mRNA/LNP or protein vaccines.

	Prefusion F ELISA GMT (95% CI)^a^	Postfusion F ELISA GMT (95% CI)^a^	RSV-A SN^b^ NT_50_^c^ GMT (95% CI)^a^
mF	2.8E + 07 (2.2E + 07, 3.6E + 07)	2.4E + 07 (1.8E + 07, 3.3E + 07)	4.4E + 04 (2.4E + 04, 7.9E + 04)
sF	2.4E + 07 (1.5E + 07, 3.7E + 07)	7.2E + 06 (5.5E + 06, 9.4E + 06)	3.2E + 04 (1.5E + 04, 6.8E + 04)
mDS-Cav1	3.8E + 07 (2.4E + 07, 5.9E + 07)	4.8E + 06 (2.3E + 06, 1.0E + 07)	7.6E + 03 (2.1E + 03, 2.7E + 04)
sDS-Cav1	1.5E + 07 (1.0E + 07, 2.2E + 07)	2.4E + 06 (2.0E + 06, 3.0E + 06)	4.9E + 03 (1.0E + 03, 2.3E + 04)
RSV F5	1.8E + 07 (9.9E + 06, 3.1E + 07)	5.4E + 06 (2.5E + 06, 1.2E + 07)	1.4E + 04 (9.3E + 03, 2.1E + 04)
RSV F7	2.6E + 07 (1.7E + 07, 3.9E + 07)	9.7E + 06 (6.6E + 06, 1.4E + 07)	1.3E + 04 (5.6E + 03, 4.4E + 04)
RSV F8	1.7E + 07 (1.1E + 07, 2.6E + 07)	4.2E + 06 (2.8E + 06, 6.2E + 06)	2.1E + 04 (9.7E + 03, 4.4 + 04)
DS-Cav1 protein + Adju-Phos ®	1.0E + 06 (1.9E + 05, 5.2E + 06)	2.4E + 05 (4.3E + 04, 1.3E + 06)	2.9E + 03 (2.3E + 02, 3.7E + 04)
Naïve	<100	<100	<4

^a^GMT (95% CI): Geometric mean of the interpolated end point titers and 95% confidence intervals.

^b^RSV-A-SN: serum neutralization of RSV-A (Long).

^c^NT_50_: titer needed to achieve 50% neutralization of RSV-A (Long).

**Fig. 2:**
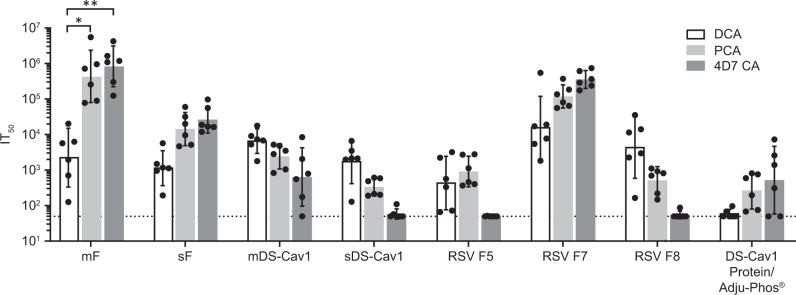
mRNA/LNP vaccines elicit conformation-indicating antibody titers in mouse. BALB/c mice (*N* = 5–6) were immunized twice with 10 μg mRNA/LNP vaccine or DS-Cav1 protein formulated with Adju-Phos®. Sera taken 2 weeks following the second immunization was assayed for the presence of D25-competing antibodies (DCA, (gray bar), palivizumab-competing antibodies (PCA, (striped bar), or 4D7 competing antibodies (4D7 CA, (white bar)). The geometric mean antibody titer needed to inhibit 50% of mAb (D25, palivizumab, or 4D7) binding to RSV F protein (IT50) is shown, along with the 95% confidence interval (CI). The dotted line indicates the limit of detection of the competition ELISA. DCA, PCA and 4D7 competing titers were compared within each group by one-way ANOVA using Graph Pad Prism software. Differences between titers within each immunization group are shown (*p* < 0.05 = *, *p* < 0.01 = **).

To assess the conformation of the expressed protein following immunization, we evaluated the ability of immune sera from vaccinated mice to compete with the palivizumab, D25, or 4D7 monoclonal antibodies for binding to RSV F protein as described.^38–40^ Competition ELISA titers were determined for mice vaccinated with mF, sF, mDS-Cav1, sDS-Cav1, RSV F5, RSV F7, and RSV F8 mRNA vaccines as well as for DS-Cav1 protein + Adju-Phos® (Fig. 2). Palivizumab binds to an epitope present in both prefusion and postfusion forms of RSV F protein and therefore, like total IgG titers, provides a measure of the overall immune response to each mRNA expressed antigen. In contrast, D25 and 4D7 specifically recognize the prefusion and postfusion conformations of F protein, respectively. Overall, mRNA vaccines expressing native forms of RSV F (mF, sF, and RSV F7) elicited modestly but not significantly higher PCA titers than and comparable DCA titers to the prefusion stabilized vaccines, possibly indicating relatively higher expression levels of these antigens in vivo. These mRNAs elicited overall higher 4D7 competing antibody titers, suggesting that a greater fraction of the expressed protein was in the postfusion conformation; however the presence of DCA in animals immunized with non-stabilized form of RSV-F suggests that the expressed antigens may transition from prefusion to postfusion conformations. In contrast, the sDS-Cav1, mDS-Cav1, and RSV F8 mRNAs, which include mutations designed to stabilize the prefusion conformation of RSV F, have higher DCA and relatively lower 4D7 competing antibody titers relative to PCA titers, supporting the interpretation that these mRNAs express RSV F protein that is predominantly in the prefusion conformation. Finally, the RSV F5 mRNA, which does include prefusion stabilizing mutations elicits an intermediate competing antibody titer, with all titers close to the limit of detection in the respective assays, suggesting that the protein is poorly expressed in mice. Interestingly, immunization with DS-Cav1 protein/Adju-Phos® resulted in relatively low DCA and relatively high 4D7 competing antibody titers. This was unexpected given that in vitro characterization of the protein antigen prior to immunization showed strong D25 binding and poor 4D7 binding (Supplementary Fig. 2). The sDS-Cav1 mRNA/LNP vaccine may elicit relatively higher D25 competing antibody titers relative to the DS-Cav1 protein because it is presented to the immune system immediately following translation and therefore may experience less loss of prefusion conformation following stress associated with purification, formulation, and routine handling prior to injection into the mouse compared with the protein antigen.

### mRNA/LNP vaccines elicit robust T cell immunity in mice

**Fig. 3:**
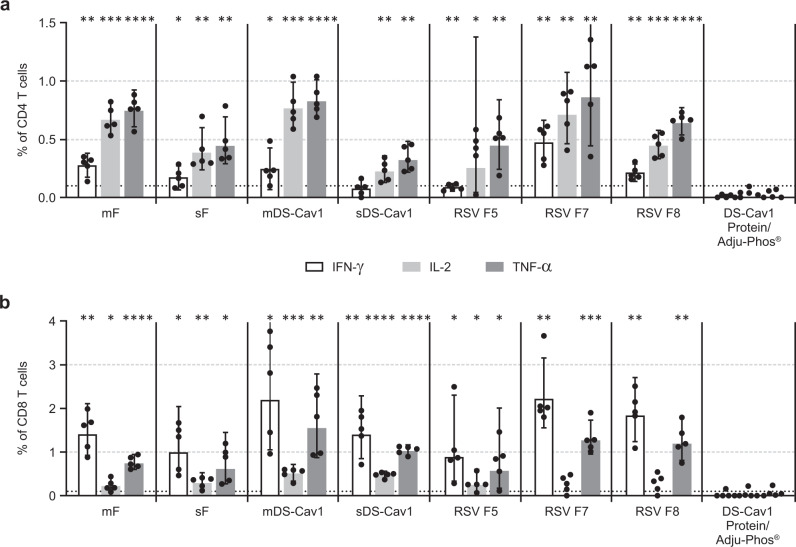
mRNA/LNP vaccines expressing RSV F elicit cellular immune responses in BALB/c mice. BALB/c mice (*N* = 5–6) were immunized twice with 10 μg mRNA/LNP vaccine or DS-Cav1 protein formulated with Adju-Phos®. Spleens were harvested 4 weeks following the second immunization. Splenocytes were incubated with pooled RSV F peptides and anti-mouse CD28 and were then stained with antibodies to the cell surface markers CD3, CD4, and CD8, as well as with a panel of anticytokine antibodies. The fluorescently tagged cells were then analyzed by flow cytometry using the gating strategy summarized in Supplementary Fig. 3. The percent of CD4+ T-cells (**a**) and CD8+ T-cells (**b**) responding to RSV F peptides with production of IFN-γ (white bar), IL-2 (light gray bar), or TNF-α (dark gray bar) is shown. Measurements for each animal are indicated (•). The height of the bar in the graph indicates the geometric mean calculation for the group ± 95% CI. *p < 0.05, **p < 0.01, ***p < 0.001, ****p < 0001 compared with the DS-Cav1 protein group by two-sided unpaired *T*-test with Welch’s correction. The dark gray dotted line indicates the limit of detection of the assay.

T cell immune responses in mice immunized with mRNA/LNP vaccines were also evaluated. Four weeks after the second immunization in the experiment described above, splenocytes were obtained and analyzed by intracellular cytokine staining (ICS). As shown in Fig. 3, all mRNA vaccines elicited F-specific CD4+ and CD8+ T cell responses, as measured by expression of IL-2, IFN-γ or TNF-α. In contrast, animals immunized with DS-Cav1 protein demonstrated very low to undetectable T cell responses (Fig. 3). Although the incorporation of prefusion stabilizing mutations did not impact the cellular immune response to the vaccine, the mF and mDS-Cav1 mRNA/LNP vaccines elicited significantly higher CD4+ T cell expression of IL-2 and TNF-α compared to their respective secreted forms (sF versus mF, *p* = 0.024 for IL-2, *p* = 0.024 for TNF-α; sDS-Cav1 versus mDS-Cav1, *p* = 0.0007 for IL-2, *p* = 0.0002 for TNF-α by unpaired *T*-test with Welch’s correction).

### mRNA/LNP vaccines protect cotton rats against RSV-A and -B challenge

**Fig. 4:**
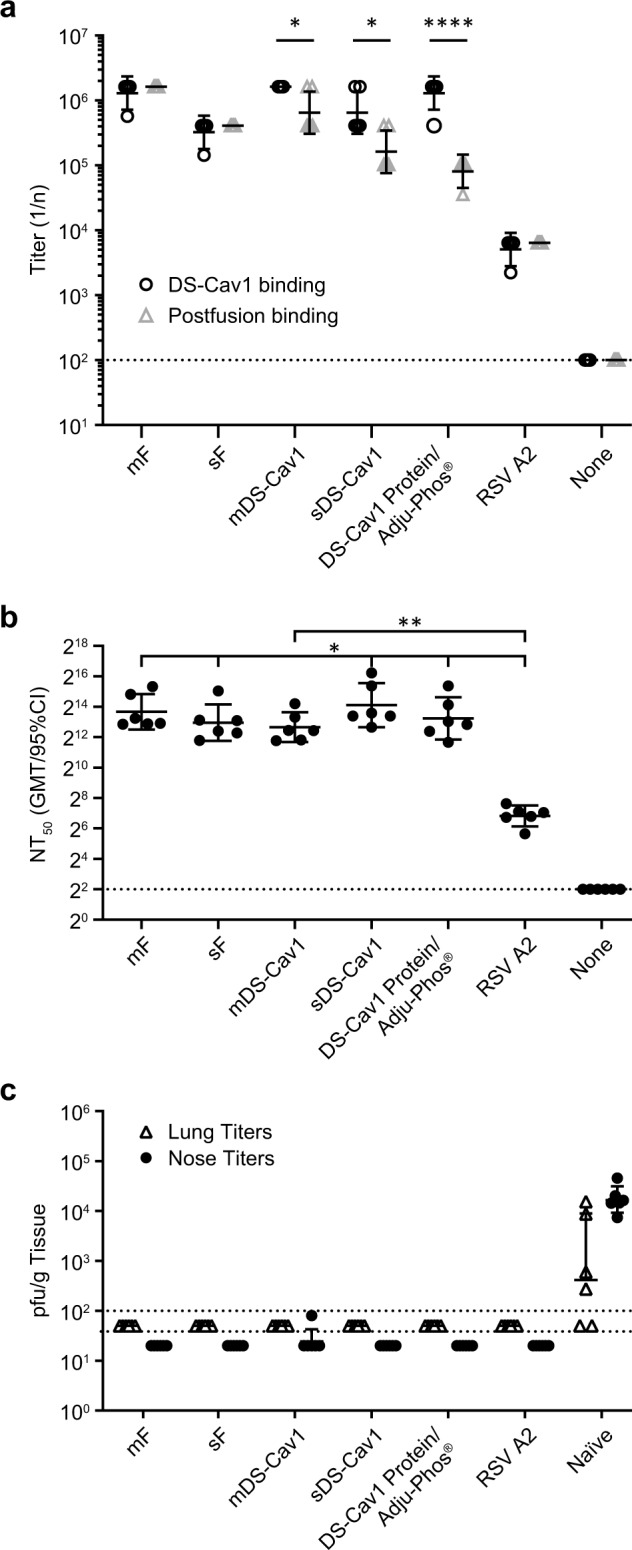
mRNA/LNP vaccines expressing RSV F are immunogenic and protective against RSV-A challenge in cotton rats. Cotton rats (*N* = 6) were immunized twice with 25 μg mRNA/LNP vaccine or DS-Cav1 formulated with Adju-Phos®. **a** Sera collected 2 weeks following the second immunization were assayed for binding to the prefusion DS-Cav1 protein (white circle) or postfusion F protein (white triangle) by ELISA. Endpoint titers are shown for each animal and the center bar indicates the geometric mean and error bars show the 95% confidence interval for each group. The dotted line indicates the limit of detection for the assay. Binding titers to prefusion versus postfusion F protein were compared for each group by two-sided unpaired t-test using GraphPad Prism software. Cotton rats immunized with mDS-Cav1, sDS-Cav1, or DS-Cav1 protein had significantly higher binding to the DS-Cav1 prefusion F protein (*p* = 0.01, 0.04, <0.0001, respectively). **b** Sera from each animal was assayed for the ability to neutralize RSV Long infection in vitro. The 50% neutralizing titer (NT_50_) was defined as the reciprocal of the serum dilution required to neutralize 50% of input virus as determined by four-parameter sigmoidal curve fit. Measurements for each animal are indicated (black circle). The center bar indicates the geometric mean titers and error bars show the 95% confidence interval. The limit of detection titer of 4 is indicated by a dashed line. Neutralizing titers for each immunized group were compared to the control group given RSV A2 intranasally by two-sided unpaired *t*-test using GraphPad Prism software and significant differences are shown on the graph. Immunization with mRNA vaccines or DS-Cav1 protein led to significantly higher neutralizing antibody titers than intranasal RSV A2 (sDS-Cav1, p = 0.04; mDS-Cav1 *p* = 0.0093; sF, *p* = 0.04; mF, *p* = 0.02; DS-Cav1 protein, p = 0.04). No other significant differences between vaccinated groups were identified. **c** Animals were challenged with RSV A2 4 weeks after the second immunization. Nose and lung tissue were collected 4 days following challenge and assayed for the presence of RSV by plaque assay. Lung titers (∆) and nose titers (•) are shown for each animal. Geometric mean titer with 95% CI is shown for each group. The assay limit of detection (LOD) is 100 for lung titers and 40 for nose titers; each is indicated on the graph with a dotted line. Animals with no RSV infection measured were plotted at one-half the LOD. Because the viral titers in the lungs of the unvaccinated, RSV-challenged animals were lower than expected, qPCR to measure RSV RNA levels was conducted on the animals in this study. The qPCR data confirmed that the animals received an effective RSV challenge and the mRNA vaccines reduced RSV titer in the lung and nose, comparable to the RSV A2 group (Supplementary Fig. 4).

The cotton rat (*Sigmodon hispidus*) is semi-permissive for RSV infection and has been used to model RSV vaccine-associated enhanced respiratory disease (VERD) and to evaluate candidate RSV vaccines for immunogenicity, efficacy and safety.^41,42^ We selected the mRNA vaccine candidates encoding sF, mF, sDS-Cav1, and mDS-Cav1 for further characterization in this model to allow additional characterization of the transmembrane and secreted forms of the DS-Cav1 antigen, in comparison with unmodified RSV F transmembrane and secreted forms. mRNA/LNP vaccines were administered intramuscularly to cotton rats in two immunizations 4 weeks apart. Additional groups of cotton rats were immunized once intranasally with RSV-A2 as a positive control for complete protection against challenge, or twice with DS-Cav1 protein to allow a comparison between the protein- and mRNA-based vaccine. All four mRNA vaccines elicited high levels of serum antibodies to RSV F proteins (Fig. 4a) along with serum neutralizing antibody titers (Fig. 4b). All of the mRNA vaccines and the DS-Cav1 protein vaccine elicited significantly higher neutralizing antibody titers compared with intranasal administration of RSV A2. Animals vaccinated with prefusion forms of RSV F (mDS-Cav1, sDS-Cav1, or DS-Cav1 protein) had significantly higher binding titers for the DS-Cav1 prefusion F protein relative to postfusion F binding titers (*p* = 0.01, *p* = 0.04, *p* < 0.0001 by unpaired *t*-test). Neutralization titers from animals immunized with RSV F-expressing mRNA vaccines were comparable to DS-Cav1 protein. Vaccinated animals were challenged with RSV A2 and lung and nasal tissue was collected 4 days post challenge. All cotton rats immunized with mRNA/LNP vaccines were protected from RSV A2 challenge in both nose and lung (Fig. 4c).

**Fig. 5:**
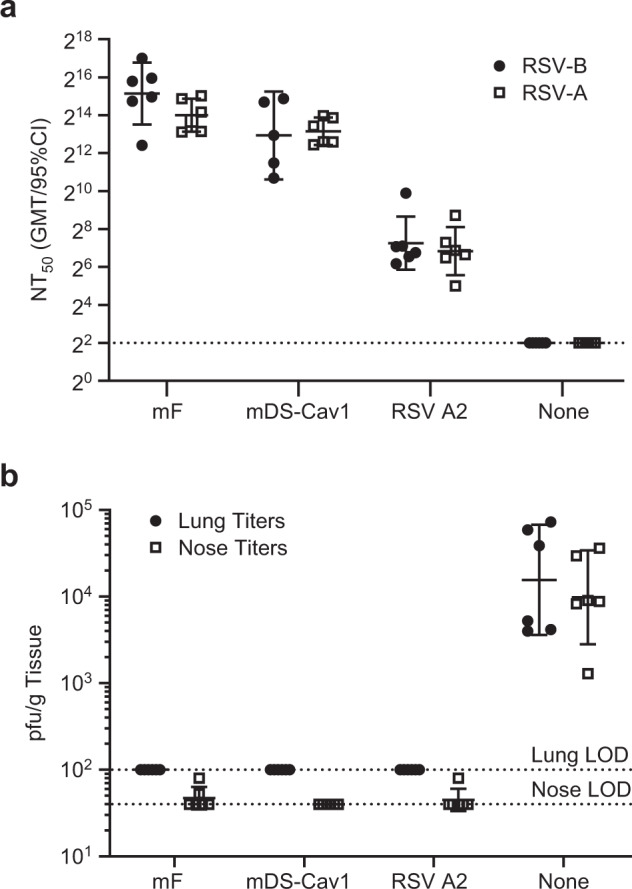
mRNA vaccines expressing RSV mF and mDS-Cav1 neutralize both RSV A and RSV B and are protective against RSV B challenge in cotton rats. Cotton rats (*N* = 6) were immunized twice with 25 μg mRNA/LNP vaccine or DS-Cav1 formulated with Adju-Phos®. Sera was isolated 4 weeks following the second immunization and assayed for neutralization of RSV A (Long) and RSV B (18537). **a** NT50 titers calculated as described above for RSV-A (Long) (white square) and RSV-B (18537) (black circle) are plotted for each group. The center bar indicates the geometric mean titers and error bars show the 95% confidence interval. The limit of detection titer of 4 is indicated by a dashed line. No difference between NT50 titers for RSV-A and RSV-B was detected for any of the groups by two-sided paired t-test. **b** Animals were challenged with RSV B 18537 4 weeks after the second immunization. Nose and lung tissue were collected 4 days following challenge and assayed for the presence of RSV by plaque assay. Lung titers (black circle) and nose titers (white square) are shown for each animal. Geometric mean titer with 95% CI is shown for each group. The assay limit of detection (LOD) is 100 for lung titers and 40 for nose titers; each is indicated on the graph with a dotted line. Animals with no RSV infection measured were plotted at the LOD.

In order to provide meaningful efficacy, an RSV vaccine must protect against disease caused by both RSV-A and RSV-B strains. The mRNA antigens used in these studies were designed based on RSV-A sequences, and although the F protein is conserved between RSV-A and -B genotypes, cross-protection should be demonstrated experimentally. To evaluate the ability of candidate vaccines to protect against RSV-B challenge, cotton rats immunized with mF or mDS-Cav1 mRNA/LNPs were challenged with RSV-B (18537) following two immunizations in comparison with RSV A2 infected, or unimmunized animals. Cotton rats immunized with either mF mRNA/LNP or mDS-Cav1 mRNA/LNP had neutralizing antibody titers against RSV-B (18537) that were comparable to the titers observed against RSV-A (Long) (Fig. 5a). The animals were completely protected from both RSV-B (18537) challenge with little to no detectable virus present in either the nose or the lung 4 days after challenge (Fig. 5b). The degree of protection was similar to that observed in animals previously inoculated with RSV A2.

### Competition ELISAs in cotton rats

**Fig. 6:**
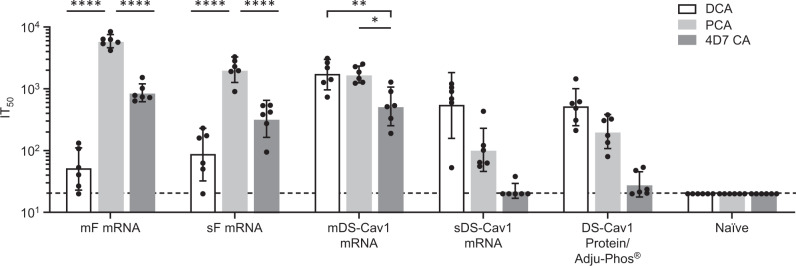
mRNA vaccines expressing prefusion RSV F elicit higher DCA titers than unmodified full length or secreted forms of RSV F in cotton rats. Cotton rats (*N* = 6) were immunized twice with 25 μg mRNA/LNP vaccine or DS-Cav1 formulated with Adju-Phos®. Sera collected 2 weeks following the second immunization were assayed for DCA (white bar), PCA (light gray bar), or 4D7CA (dark gray bar). The IT50 for each animal is plotted, and the geometric mean and 95% confidence intervals is shown. The dotted line at IT50 = 20 indicates the LOD for the assay. Animals with no competing antibody titers were assigned a titer at the LOD. DCA, PCA and 4D7 competing titers were compared within each group by one-way ANOVA using Graph Pad Prism software. Differences between titers within each immunization group are shown (*p* < 0.05 = *, *p* < 0.01 = **, *p* < 0.001 = ***, *p* < 0.0001 = ****).

In order to confirm that the mRNA vaccines resulted in the expression of RSV F proteins of the expected conformation, the ability of sera from vaccinated cotton rats to compete with the antibodies palivizumab, D25, and 4D7 was evaluated as described above (Fig. 6). As observed in the mouse study above, all forms of RSV F evaluated elicited PCA and DCA, and the mRNAs expressing prefusion stabilized forms of RSV F (sDS-Cav1 and mDS-Cav1) elicited the highest DCA titers, while non-stabilized forms of RSV F (mF and sF mRNAs) elicited the lowest DCA titers. 4D7 competing antibody titers were lower overall in cotton rats than in BALB/c mice. Surprisingly, the mDS-Cav1 mRNA elicited 4D7 titers that were comparable to the mF and sF mRNAs, while 4D7 titers following vaccination with sDS-Cav1 were at the lower limit of detection in the assay. mRNAs expressing forms of RSV F with prefusion stabilizing mutations elicited higher DCA titers relative to PCA titers.

### mRNA/LNP vaccination does not lead to VERD in cotton rat

A cotton rat study was conducted to assess the potential for the mF and mDS-Cav1 mRNA/LNP vaccines to promote VERD in comparison to the original Lot 100 FI-RSV vaccine, which was linked to VERD in a clinical trial in RSV naïve infants and toddlers.^42^ Groups of 10 cotton rats were given two immunizations three weeks apart with mF mRNA/LNP, mDS-Cav1 mRNA/LNP, a control Luciferase mRNA/LNP, an LNP only control, the original Lot 100 FI-RSV, a more recently generated FI RSV prepared using similar methods to the Lot 100 vaccine, or no vaccine. Four weeks following the second immunization, the cotton rats were challenged with RSV A2. One group of unvaccinated animals was not challenged as a control. Five days after challenge, the animals were euthanized. The nose of each cotton rat was processed for recovery of virus and titers were determined for each animal. The lungs from each animal were trisected and processed for viral titration, qPCR, or histological examination after fixation in formalin. The formalin-preserved lung from each cotton rat was processed and examined for evidence of pathology.

As observed previously (Fig. 4), the mF and mDS-Cav1 mRNA/LNP vaccines were immunogenic, eliciting robust RSV F-binding ELISA and serum neutralizing titers. In contrast, the control groups, including those animals immunized with Lot 100 FI-RSV had no detectable serum RSV-neutralizing antibody titers (Supplementary Fig. 5a). Similarly, animals immunized with mF mRNA/LNP or mDS-Cav1 mRNA/LNP were completely protected from RSV challenge, while all other RSV-challenged groups had an average above 30,000 pfu/g of RSV recovered from the nose. Animals vaccinated with mF mRNA/LNP or mDS-Cav1 mRNA/LNP were completely protected from RSV challenge in the lung as well. Animals immunized with luciferase/LNP or LNP alone had 16,000 and 20,000 pfu of RSV per gram of lung tissue, similar to unimmunized and challenged cotton rats, while those immunized with FI-RSV showed partial protection (Supplementary Fig. 5b, c).

**Fig. 7:**
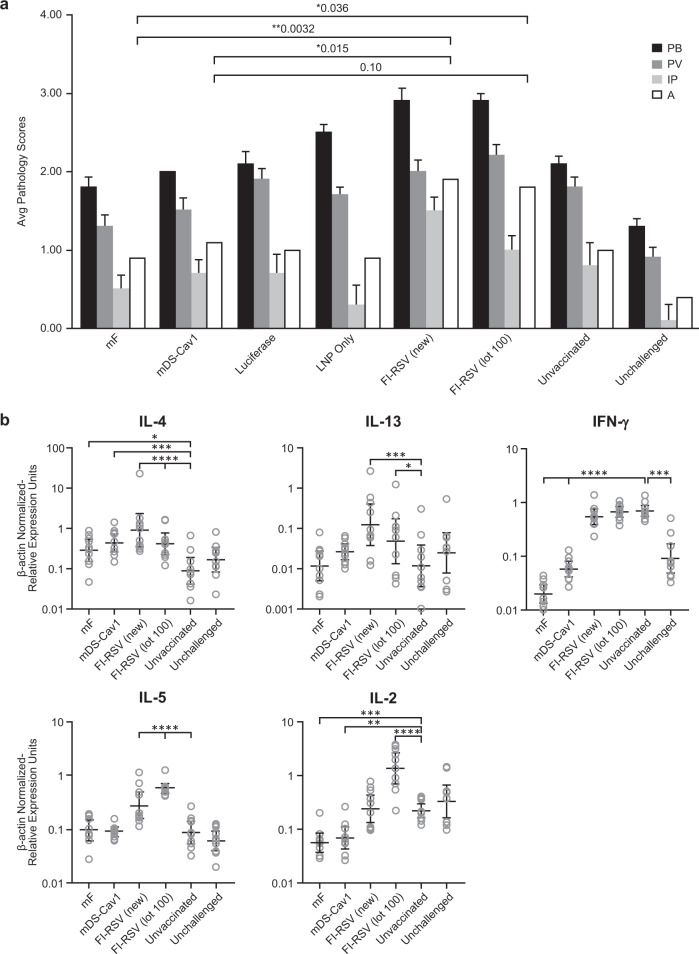
RSV F-mRNA Vaccines do not lead to VERD in cotton rat. Cotton rats (*N* = 10) were immunized twice with 25 μg mF mRNA/LNP, mDS-Cav1 mRNA/LNP, Luciferase mRNA/LNP, LNP only (no RNA), FI-RSV (lot 100), FI-RSV generated using similar methods to lot 100 (FI-RSV (new)) or were left unvaccinated. With the exception of one unvaccinated group, all groups were challenged 4 weeks following the second immunization with RSV A2. Five days following the challenge, the animals were sacrificed. Lungs from each animal were trisected and processed for virus quantification (Supplementary Fig. 5b, c), pathology (**a**), and cytokine mRNA analysis (**b**). **a** Lung pathology following RSV challenge. Lung tissue from each animal was embedded in paraffin, sectioned, stained with hematoxylin and eosin, and given a pathology score from 0 (no pathology) to 4 (severe pathology) by a blinded pathologist. Sections were scored for peribronchiolitis (PB), perivasculitis (PV), interstitial pneumonia (IP), and alveolitis (A). Mean pathology score with standard error is shown. Raw histopathology scores for each animal are shown in Supplementary Table 1. Lung pathology scores were assessed using a two-sided exact permutation test. The mF and mDS-Cav1 mRNA vaccines resulted in significantly lower levels of pathology relative to both FI-RSV controls (for alveolitis, *p* = 0.0032 for mF versus FI-RSV (new) and 0.0358 for mF versus FI-RSV lot 100; *p* = 0.0151 for mDS-Cav1 versus FI-RSV (new) and 0.1016 for mDS-Cav1 versus FI-RSV lot 100), and the pathology scores for these two groups are not significantly different from the luciferase mRNA/LNP, LNP alone, unvaccinated and unchallenged negative control groups. **b** Cytokine gene expression after vaccination and RSV challenge. mRNA was isolated from the lungs of each cotton rat and IL-4, IL-13, IL-5, IFN-γ, and IL-2 mRNA levels were determined by rtPCR. The relative level of each mRNA was determined by normalizing to levels of the housekeeping gene β-actin and plotted for each animal. The geometric mean and 95% confidence interval for the β-actin normalized relative mRNA expression units for each of the five cytokines is shown. Differences between the groups were determined by two-sided *t*-test using the error term from a one-way ANOVA. Significant differences from unvaccinated, challenged animals are shown on the graph, with *p* < 0.05 = *, *p* < 0.01 = **, *p* < 0.001 = ***, and *p* < 0.0001 = ****. Relative to the animals in the two FI-RSV groups, animals immunized with mF or mDS-Cav1 had lower mRNA expression of IFN-γ (for both mF and mDS-Cav1, *p* < 0.0001 for both FI-RSV (new) and FI-RSV Lot 100), lower expression of IL-2 (for mF, *p* < 0.0001 for FI-RSV (new) and *p* < 0.0001 for FI-RSV (lot100); for mDS-Cav1, *p* = 0.0006 for FI-RSV (new) and *p* < 0.0001 for FI-RSV (lot100)); lower expression of IL-13 (for mF, *p* = 0.0008 versus FI-RSV (new) and 0.0375 for FI-RSV (lot100); for mDS-Cav1 *p* = 0.0236 versus FI-RSV (new) and *p* = 0.3625 (no difference) for FI-RSV (lot100)); and lower expression of IL-5 (*p* < = 0.0001 comparing either mF or mDS-Cav1 with either FI-RSV (new) or FI-RSV (lot100). IL-4 expression was significantly decreased for mF only when compared to FI-RSV (new) (*p* = 0.0118), while mDS-Cav1 showed a trend toward significance (*p* = 0.1045). The overall pattern of cytokine gene expression in the lungs of mF and mDS-Cav1 mRNA/LNP immunized cotton rats is most similar to the Group 8 unvaccinated and unchallenged animals, which reflects the complete protection from RSV challenge observed in these animals (Supplementary Fig. 5b, c).

Mean pathology scores for each treatment group are shown in Fig. 7a. Each lung was scored blindly for each finding, with a severity score of 0–4, with 0 being no evidence of pathology and 4 having the most pathology. Each lung was evaluated for evidence of peribronchiolitis, perivasculitis, interstitial pneumonia, or alveolitis, with the latter two pathologies being the two predominant observations defining VERD in animals immunized with FI-RSV. Lung pathology scores were assessed using a two-sided exact permutation test. Animals vaccinated with mF mRNA/LNP or with mDS-Cav1 mRNA/LNP showed alveolitis levels that were similar to those seen in animals within the unimmunized challenge control group (*p* = 0.7116 and *p* = 1.0000 for the raw scores; *p* = 0.8096 and p = 1.0000 for the pathological scores). Animals vaccinated with the FI-RSV Lot 100 vaccine preparations showed the highest alveolitis scores with the differences between these two groups and the other four groups approaching statistical significance (*p* ≤ 0.10). Similar observations were made regarding interstitial pneumonitis, though with somewhat lower severity scores and a correspondingly lesser degree of statistical significance.

### mRNA/LNP vaccination does not lead to upregulation of Th2 cytokines in RSV-challenged cotton rat

Polarization of the CD4+ T-cell response to a Th2 phenotype following FIRSV vaccination has been linked to VERD in mouse models (reviewed in refs. ^43,44^), so we elected to evaluate cytokine expression in lung tissue obtained from cotton rats in the above study. mRNA was recovered from the lung of each cotton rat and was evaluated for the expression of Th1 and Th2-associated cytokines including IFN-γ, IL-2, IL-4, IL-5, and IL-13. Differences between groups in lung cytokine mRNA expression were evaluated using the natural log transformed, β-actin mRNA normalized relative expression units by two-sided t-test using the error term from a one-way ANOVA (Fig. 7b). As expected for an ERD-associated Th2 response, expression of mRNA for IL-2, IL-4, IL-5, and IL-13 was highest in groups vaccinated with the FI-RSV preparations. Overall, induction of these cytokines in animals immunized with mF and mDS-Cav1 vaccines were lower relative to the other groups, consistent with these groups showing protection from RSV challenge.

## Discussion

mRNA/LNP vaccination is an emerging approach that has demonstrated the ability to elicit strong immune responses in rodents, rabbits, non-human primates, and in humans.^29,30,33,35,45–47^ The technology provides many of the advantages achieved using viral vector vaccine platforms, including the potential to elicit cellular immune responses, and the ability to express both membrane-associated and secreted antigens, as well as antigens that are difficult to purify or have poor stability. In contrast to viral vectored vaccines where anti-vector immune responses may limit the ability to utilize the platform for boost immunizations, mRNA vaccines can be delivered multiple times without immune interference, and mRNA vaccine candidates can be produced at higher throughput.

RSV F protein is an ideal antigen for demonstrating the value of the mRNA/LNP platform, because of the labile nature of the more immunogenic prefusion conformation of the antigen. Since the failure of the purified F protein (PfP) and F/G/M RSV vaccines, which were based on RSV F or multi-antigen preparations extracted directly from RSV, a number of groups have explored engineered forms of RSV F protein designed to improve the stability and/or immunogenicity of the antigen.^7,8,11,13,44,48^ For recombinant protein-based vaccines in general, the resulting immunogenicity of the antigen is intrinsically related to the cell line used to express the antigen, as well as the methods used to purify the antigen, which will impact the nature of post-translational modifications to the antigen and/or the conformation of the antigen, respectively.^49^ Nucleic acid vaccination may allow for an improved immune response to conformation dependent antigens such as RSV F because the vaccine antigen is translated by host cells and is then presented to the immune system immediately upon translation. Here we used a mRNA/LNP vaccine platform to compare different forms of RSV F in rodent models and showed that these vaccines were both immunogenic and protective against RSV A and B challenge.

The forms of RSV F protein that were tested as mRNA vaccines included both full length and ectodomain forms, monomeric and trimeric, prefusion stabilized and nonstabilized forms of the antigen. Little difference was observed in the serum neutralizing antibody produced following immunization with full length compared with ectodomain forms of RSV F (mF versus sF; mDS-Cav1 versus sDS-Cav1, Tables 2 and 4, and Fig. 4b). We initially evaluated a number of RSV F antigens that included mutations designed to stabilize RSV F in its prefusion conformation, but then focused our analysis of prefusion stabilized antigens on those containing the well-characterized DS-Cav1 mutations.^7^ Somewhat surprisingly, the neutralizing antibody titers elicited by prefusion stabilized versus non-stabilized forms of RSV F (mF versus mDS-Cav1 and sF versus sDS-Cav1) were not improved by incorporation of the DS-Cav1 mutations in either mouse or cotton rat (Table 4 and Fig. 4b). One possibility for this observation may be that following translation of wild type forms of RSV F, the antigen initially achieves a prefusion conformation long enough to elicit antibody responses. This possibility is supported by the observation of DCA titers in mice and cotton rats immunized with mF and sF mRNAs, and the ability of D25 to bind to cells expressing the mF mRNA (Table 4, Fig. 6). The observation of higher DCA:PCA ratios in mouse and cotton rat immunized with mDS-Cav1 and sDS-Cav1 compared with mF and sF suggests that the mDS-Cav1 and sDS-Cav1 antigens are stabilized in the prefusion conformation in vivo as designed.

The incorporation of competitive antibody binding assays for prefusion and postfusion conformation-indicating antibodies resulted in some surprising observations. In addition to the common practice of measuring levels of palivizumab competing antibodies, we assayed sera from immunized mice and cotton rats for antibodies that compete with D25 (site Φ) for binding to the DS-Cav1 prefusion F protein and for antibodies that compete with 4D7 which binds to antigenic site I in the postfusion form of RSV F.^36^ In both mice and cotton rats, we observed that mRNA vaccines expressing forms of RSV F lacking prefusion stabilizing mutations elicited higher 4D7 competing antibody titers, while mRNA vaccines expressing prefusion stabilized RSV F had higher D25 competing antibody titers relative to overall antigen expression as determined by PCA. We also observed antigen-specific differences between mouse and cotton rat immune responses. Mice immunized with DS-Cav1 protein had low DCA titers and higher 4D7 competing titers, while cotton rats immunized with DS-Cav1 protein/Adju-Phos® had high DCA titers and low 4D7 competing titers, suggesting the potential for greater sensitivity to RSV antigenic site I in the BALB/c mouse model. In both mice and cotton rats, we found that the mDS-Cav1 mRNA vaccine elicits both DCA and 4D7 competing antibodies in mice and cotton rats, suggesting either that the mDS-Cav1 mRNA is translated into a mixed population displaying antigenic epitopes associated with both prefusion and postfusion conformations, or that the membrane-associated prefusion form of F protein maintains antigenic site I to a greater degree than the soluble form.

The use of the mRNA platform for immunization may provide additional benefit in improving the cellular immune response to vaccination relative to vaccination with a protein antigen. We observed a robust CD4+ and CD8+ T cell response to RSV F peptides in all mice immunized with mRNAs expressing forms of RSV F. The different forms of RSV F mRNA vaccines led to roughly equivalent cellular immune responses, although the membrane-associated forms of wild type and DS-Cav1 RSV F did elicit higher CD4+ T cell responses than the secreted forms of these antigens. Importantly, the DS-Cav1 protein/Adju-phos® vaccine elicited little to no cellular immunity in mice (Fig. 3). The observed lack of CD4 response to DS-Cav1 protein/Adju-phos® compared with mRNA vaccines expressing similar antigens (sDS-Cav1 or mDS-Cav1) may underlie the generally lower quality humoral response observed to the protein-based vaccine in mice, where neutralizing antibody and DCA titers were reduced relative to vaccination with the corresponding sDS-Cav1/LNP mRNA vaccine (Fig. 2 and Table 4).

Overall, a comparison of the different forms of RSV F evaluated reveals that there were differences in the quality of the immune response elicited by the different forms of RSV F. Antigens including the prefusion stabilizing DS-Cav1 mutations elicited higher DCA titers relative to either PCA or 4D7 competition ELISA titers, indicating that more of the neutralizing antibody response elicited by these antigens is directed against antigenic site ∅. As antigenic site ∅ is predominant in the natural human immune response to RSV, it may be preferable to select an antigen containing the DS-Cav1 mutations, which support greater presentation and stabilization of this epitope compared to wild type RSV. Despite the increased in DCA in mDS-Cav1 and sDS-Cav1, the neutralizing antibody titer elicited in mice and cotton rats was not increased relative to mF or sF in mice or cotton rats. This may be due to differences in antigen expression in vivo, or the mutations incorporated into the antigens to stabilize the prefusion conformation may either impact the overall stability of the molecule or may not completely lock in the prefusion conformation in vivo. Although the mDS-Cav1 mRNA elicited higher 4D7 competing antibody titers than sDS-Cav1 in both cotton rats and mice, the mDS-Cav1 mRNA vaccine also elicited significantly higher CD4+ T-cell responses in mice than the sDS-Cav1 mRNA, which should be considered when selecting antigens for further evaluation.

A significant challenge for the development of RSV vaccines is the need to demonstrate vaccine safety. Early studies evaluating FI-RSV vaccination in naïve infants established the potential for VERD leading to significant increases in hospitalization and death following RSV infection.^43^ The cotton rat has been widely used to evaluate candidate vaccines for safety prior to clinical testing. We demonstrated that our candidate mRNA vaccines do not carry the same safety liabilities as FI-RSV. Although lung pathology was observed in the unvaccinated, RSV-challenged animals following RSV challenge, cotton rats vaccinated with mF mRNA/LNP or mDS-Cav1 mRNA/LNP and subsequently challenged with RSV had lung pathology scores that were not significantly different than unimmunized animals. Importantly, alveolitis scores were significantly lower than those recorded for FI-RSV immunized animals. As VERD following RSV infection has been associated with a Th2 biased immune response, we assessed cytokine gene expression in the lungs of the vaccinated and RSV-challenged animals.^43^ As described by others, IFN-γ was upregulated in both unvaccinated animals and in those vaccinated with FI-RSV following RSV challenge, correlating with RSV infection; in contrast the expression of the Th2-associated cytokines IL-4, IL-5, and IL-13, was upregulated in animals vaccinated with FI-RSV, but not in unvaccinated controls.^50^ In contrast, cytokine expression in the lungs of cotton rats vaccinated with mF or mDS-Cav1 mRNA/LNP vaccines and then challenged with RSV is significantly diminished compared with FI-RSV immunized animals and appears most similar to animals that were neither vaccinated nor RSV-challenged. The data suggest that both mRNA vaccines prevented RSV infection and the accompanying inflammatory response associated with infection; however, to build more confidence in vaccine safety, the vaccine ultimately selected for clinical evaluation will need to be evaluated for VERD induction in cotton rats following immunization using a suboptimal vaccine dose prior to testing in seronegative infants or toddlers.

In summary, we have evaluated mRNA vaccines expressing multiple forms of RSV F in mouse and cotton rat models. While mRNA vaccines expressing both prefusion stabilized and wild type forms of RSV F protein elicit robust neutralizing antibodies and equivalent levels of protection in cotton rats, RSV F antigens including the DS-Cav1 prefusion stabilizing mutations elicited a greater proportion of antibodies targeting antigenic site ∅, but do not result in increased neutralizing antibody titer in mice or cotton rats. Regardless of the form of RSV F expressed, mRNA vaccines elicited significantly higher CD4+ and CD8+ T cell responses in mice compared with the DS-Cav1 protein antigen, although membrane-associated forms of RSV F do appear to elicit higher CD4 responses. Finally, mRNA vaccines expressing forms of RSV F do not lead to VERD in cotton rats. Taken together, these data support the continued evaluation of mRNA vaccines expressing RSV F as vaccine candidates for the prevention of RSV disease. Future studies will include the evaluation of newer single chain versions of RSV-F with improved prefusion stability,^9,10^ and an expansion of the evaluation of the ability of these vaccine candidates to boost the immune response to RSV in previously exposed animals as a model for adult vaccination.^51,52^

## Methods

### Materials

RSV strains A2 (ATCC VR-1540) and B strain 18537 (ATCC VR-1580) were used for challenge. RSV Long (ATCC VR-26) was used in serum neutralization assays.

RSV F-expressing mRNAs were designed based on the RSV F sequence from the RSV A2 strain, and modifications were incorporated as described in Fig. 1. To generate the mRNA vaccines, plasmid encoding the T7 RNA polymerase promotor followed by 5′ untranslated region (UTR), open reading frame (ORF), 3′UTR, and polyA tail was overexpressed in *E. coli*, linearized, and purified to homogeneity. The mRNAs were synthesized by in vitro transcription using T7 RNA polymerase, where uridine triphosphate was substituted with N1-methyl-pseudouridine triphosphate.^30^ Cap 1 was utilized to improve translation efficiency. After the transcription reaction, mRNAs were purified, buffer exchanged into sodium citrate buffer, and stored at −20 °C until use. mRNA formulations were carried out either at Merck (Figs. 2, 3, and 5) or Moderna (Figs. 4, 6, and 7). For Merck formulations, LNP encapsulating mRNA were prepared by rapid precipitation process as previously described.^53^ The lipid components of the LNP comprised an asymmetric ionizable amino lipid, 1,2-distearoyl-sn-glycero-3-phosphocholine (DSPC), cholesterol and poly(ethylene glycol)2000-dimyristoylglycerol (PEG2000-DMG) in a molar ratio of 58:30:10:2, respectively. For formulations carried out by Moderna, LNPs were generated as described in,^34^ and lipids were dissolved in ethanol at molar ratios of 50:10:38.5:1.5 (ionizable lipid: DSPC: cholesterol: PEG-lipid). All formulations were tested for particle size, mRNA encapsulation, and endotoxin prior to injection into animals.

An expression vector for the generation of DS-Cav1 protein was kindly provided by Drs. Barney Graham and Peter Kwong, VRC, NIH. Recombinant prefusion F (DS-Cav1) and postfusion F proteins were expressed and purified as described previously.^12^

The monoclonal antibody 4D7 was generated internally and is described in (Flynn 2016). To produce human antibodies D25 and AM14,^54^ the heavy and light chain variable region sequences were subcloned into a pTT5 vector. Plasmids encoding both the heavy and light chain were transiently co-transfected into suspension CHO-3E7 cells grown in serum-free FreeStyle CHO Expression Medium (Life Technologies). The supernatants collected after 6 days were applied to a Protein A CIP column (Genscript, Piscataway NJ) for purification. The purified antibodies were buffer-exchanged to phosphate-buffered saline (PBS).

D25, Palivizumab, 4D7 and AM14 antibodies were labeled using Alexa Fluor 488, Alexa Fluor 647, or biotin Labeling Kits (ThermoFisher) to a 1 µg/mL concentration.

### Cell transfection

Expi293F cells (ThermoFisher) were transfected with mRNA constructs using the ExpiFectamine 293 Tranfection Kit (ThermoFisher) protocol. Briefly, 80 µL ExpiFectamine 293 Reagent were incubated with 1.5 mL Opti-MEM I Reduced Serum Medium for 5 min at room temperature before mixing with 10 µg of mRNA in 1.5 mL Opti-MEM I Reduced Serum Medium. After 20 min incubation at room temperature, increasing amounts of this mRNA/ExpiFectamine mix (50, 100, 250, and 500 µL) were added to Expi293F cells incubated in 24-well plates (0.6 ml/well, 1.8 × 10^6^ cells/well) in Expi293 Expression Medium. Cells were placed on an orbital shaker (125 rpm) in a cell incubator at 37 °C for 24 h. At that time, cell supernatants were collected and kept at −20 °C until ELISA analysis and cells were stained for FACS analysis.

### Cell staining

After transfection, Expi293 cells (0.5 × 10^6^ cells) were pelleted in a V-bottom 96-well plate and resuspended in PBS (250 µL). Live/Dead Fixable Aqua Dead Cell stain (ThermoFisher) reconstituted in DMSO per manufacturer’s instructions was added to each well (1 µL /well) and incubated at room temperature in the dark for 30 min. Cells were pelleted, washed twice with Stain Buffer (FBS) (300 µL/well, BD Biosciences), and added to a V-bottom 96-well plate (0.5 × 10^6^ cells/well). Cells were resuspended in Stain Buffer (FBS) with D25-Alexa Fluor 488 or AM14-Alexa Fluor 488 and 4D7-Alexa Fluor 647, or isotype control antibodies (each at 5 µg/mL) and incubated for 15 min on ice in the dark. Cells were then pelleted and washed twice with Stain Buffer (FBS) (300 µL/well), resuspended in Stain Buffer (FBS) (200 µL/well), analyzed in a LSR II flow cytometer (BD Biosciences), and data generated were processed using by FlowJo software (Tree Star, Inc). A representative sample of the flow data and gating strategy are included in Supplementary Fig. 3.

### Sandwich ELISA

Cell supernatants were analyzed following standard sandwich ELISA protocols. Briefly, 96-well flat bottom plates (MaxiSorp ELISA plates, Nunc) were incubated with Palivizumab antibody in PBS (1 µg/mL, 100 µL/well) at 4 °C overnight. Plates were washed once with PBS-T (ThermoFisher) using a BioTek EL406 Washer and incubated with Block buffer (2% BSA (Bio-Rad) in PBS-T) at 37 °C for 1 h. After five washes with PBS-T, thawed cell supernatants were added to the wells at 1/5 or 1/25 dilutions (75 µL/well) in Block buffer. Purified recombinant prefusion or postfusion F proteins were added to control columns in 8 step 2-fold dilutions starting at 0.5 µg/mL concentration. After 2 h room temperature incubation, plates were washed six times with PBS-T and incubated with biotinylated D25, 4D7, or AM14 antibodies (20 µg/mL, 75 µL/well in Block buffer). After 1 h room temperature incubation, plates were washed six times with PBS-T and incubated with Streptavidin-HRP (Pierce) following manufacturer’s recommendations. After 30 min incubation, plates were washed six times with PBS-T and incubated with TMB Substrate (100 µL, ThermoFisher). Color development was stopped after 15 min with 2 M H_2_SO_4_ (100 µL) and plates were read in a Tecan Infinite M1000 pro at a 450 nm wavelength.

### Bio-layer interferometry (BLI) for qualification of DS-Cav1 protein

A Pall ForteBio Octet Red96e instrument was used to assess association between RSV F variants, sDS-Cav1 and sF, and antibodies against various RSV F conformations. A non-RSV soluble protein was included as a negative control. All assays were completed with agitation at 1000 rpm. Assays were performed at 25 °C in flat bottom black 96-well plates (Greiner Bio-One) with evaporation covers. All antibodies and proteins were diluted in 1X Kinetics Buffer (1X KB: 10X Kinetics Buffer (Pall ForteBio) diluted 1:10 in phosphate-buffered saline (PBS)). The final volume for all solutions in the plate was 200 μl/well. Anti-human IgG Fc Capture sensors (AHC: Pall ForteBio) were stabilized with 5 s alternating pulses of 10 mM glycine pH 1.75 and 1X KB for three cycles, baselined for 60 s in 1X KB and then loaded with antibodies at a concentration of 5 μg/ml for 200 s. Biosensor tips were equilibrated for 300 s in 1X KB before measurement of association with RSV F and non-RSV soluble proteins (25 nM) for 600 sec. Proteins were allowed to dissociate for 600 s. Data analysis and curve generation were completed using ForteBio Data Analysis 10.0 software. To account for systemic baseline drift, all data were background subtracted with the measurement of a reference well, an antibody-loaded sensor incubated in 1X KB buffer alone. All processed data was *y*-axis aligned to baseline.

### Mouse immunizations

Mouse studies were approved by the Institutional Animal Care and Use Committee at Merck & Co., Inc., Kenilworth, NJ, USA. Groups of 10 female BALB/c mice (CRL) aged 5–7 weeks were immunized twice at a 3-week interval with 10 μg DS-Cav1 protein formulated with Adju-phos® (InvivoGen, San Diego, CA) or with the same dose of candidate mRNA vaccines in a lipid nanoparticle formulation. Two weeks following the second immunization, blood was drawn for serological assays. For ICS assays, animals were sacrificed 4 weeks following the second immunization and spleens were harvested.

### Cotton rat immunizations

Cotton rat studies were approved by the Institutional Animal Care and Use Committee at Merck & Co., Inc., Kenilworth, NJ, USA. Groups of six female cotton rats (SAGE) aged 3–7 weeks were immunized twice intramuscularly with 25 μg mRNA/LNP vaccine or DS-Cav1/Adju-Phos® on days 0 and 28 of the study, or with a single intranasal administration of 10^5.5^ pfu RSV A2. Animals were bled for serology prior to the first immunization and on days 21, 28, 42, and 56. On day 56 (4 weeks following the second immunization), all animals in the study were challenged intranasally with 5.9 × 10^5^pfu of RSV A2 or B 18537 strains delivered in a 0.1 mL volume. Four days following the RSV challenge, animals were euthanized and lung and nose tissue were isolated for quantification of viral load.

The cotton rat VERD study was conducted at Sigmovir Biosystems (Rockville, Md). Groups of 10 female cotton rats (Sigmovir) aged 3–7 weeks were immunized with the vaccine candidates mF mRNA/LNP, mDS-Cav1 mRNA/LNP, or with the following controls: luciferase mRNA/LNP as a control for immunization with mRNA expressing a non-RSV protein, empty LNP with no mRNA, FI-RSV Lot 100 from the original FI-RSV clinical trial,^42^ a more recently generated FI-RSV, and two unvaccinated groups. One of the unvaccinated groups was challenged to serve as a control for the pathological changes associated with natural infection, while the second was left unchallenged to serve as a control for natural cotton rat physiology. The cotton rats were immunized twice intramuscularly at a 3-week interval, and all groups, with the exception of one of the unvaccinated groups which was left untreated as a control for normal cotton rat pathology, were challenged intranasally 4 weeks after the second immunization with 10^5.5^pfu of RSV A2 in 0.1 mL volume. Five days following the challenge, animals were sacrificed. Noses were harvested for virus quantification, and lungs from each animal were trisected and processed for virus quantification, pathology, and cytokine mRNA analysis.

### ELISAs

ELISAs to evaluate binding antibody titers against prefusion or postfusion F protein were conducted essentially as described in ref. ^12^. Ninety-six-well ELISA plates (NUNC) were coated with 2 μg/mL purified recombinant RSV F protein DS-Cav1 or purified recombinant postfusion RSV F protein as described in ref. ^7^ and ref. ^55^, respectively, and incubated at 4 °C overnight. The plates were then washed and blocked for 1 h at room temperature with 3% non-fat milk dissolved in PBS-T. Sera from mice or cotton rats was serially diluted 4-fold in blocking buffer, transferred to the RSV F coated plates, and incubated for 2 h at room temperature. The plates were then washed three times with PBS-T. Following the plate wash, HRP conjugated goat anti-mouse/rat IgG secondary antibody (Invitrogen), diluted at 1:3000 in blocking buffer, was added to the plates and incubated for an additional 1 h. Plates were washed again and developed with SuperBlu Turbo TMB (Virolabs). The reaction was stopped after 5 min and absorbance was read at 450 nm on a VersaMax ELISA microplate reader (Molecular Devices). Endpoint titers were defined as the reciprocal of the end point dilution at which the serum sample has an optical density (OD) signal greater than or equal to two (2×) times that of the background.

### Serum neutralization assays

Serum neutralization assays were conducted as described in ref. ^56^ Briefly, sera were heat inactivated and serially diluted into a 96-well plate. The sera was combined with RSV (Long) at a final concentration of 100 pfu/well. HEp-2 cells were added to each well and the plates were incubated at 37 °C for 72 h. Cells were then washed and fixed with acetone. Each well was then incubated with internally generated guinea pig hyperimmune sera, followed by biotinylated horse anti-guinea pig monoclonal antibody (Vector laboratories). The signal was developed by adding a cocktail of IRDye 800CW Streptavidin (Li-Cor Biosciences, 1:1000 final dilution), Sapphire 700 (Li-Cor Biosciences, 1:1000 dilution) and 5 mM DRAQ5 solution (Biostatus Ltd, 1:10,000 dilution) in assay diluent. Plates were read on an Aerius® Automated Imaging System. Titers were calculated by four-parameter curve fit using GraphPad Prism® 7 software.

### Intracellular cytokine staining

Mouse splenocytes were cultured in R10 medium (RPMI 1640 supplemented with 10% of fetal calf serum, 1 × penicillin/streptomycin, 1 mM MEM sodium pyruvate, 2mM L-glutamine, 10 mM 4-(2-hydroxyethyl)-1-piperazineethanesulfonic acid buffer, and 50 nM 2-Mercaptoethanol) at 37 °C in a 4–6% carbon dioxide incubator in round bottomed plates. 2 μg/mL concentration RSV F peptides (15 mers of the entire F sequence overlapping by 11mers, custom order JPT, Germany) and anti-mouse CD28 and CD49d costimulatory antibodies were added into each assay well at final concentrations of 2 μg/mL. The plates were incubated at 37 °C for 30–60 min. After incubation, freshly diluted brefeldin A was added, and the plates were incubated for an additional 4–5 h. Cells were washed once with FACS wash buffer (PBS with 1% FBS and 0.01% sodium azide) and stained with a cocktail of LIVE/DEAD™ Fixable Violet Stain and fluorescently-labeled antibodies specific to cell surface markers CD3 (clone 145-2C11), CD4 (RM4-5), CD8 (53-6.7) (BD Biosciences). After 15 min incubation, cells were washed and incubated with 200 µL/well BD cytofix solution (BD Biosciences) at 4 °C for 20 min, washed twice with BD perm wash buffer (BD Biosciences), and stained with a cocktail of fluorescently-labeled anti-cytokine antibodies (TNF-α clone MP6-XT22, IFN-γ clone XMG1.2, IL-2 clone JES6-5H4, IL-10 JES5-16E3, all from BD Biosciences and IL17A TC11-18H10.1, Biolegend). The cells were washed once with BD perm wash buffer and resuspended in 200 µL BD stabilizing fixative. The fluorescent signals were analyzed by an LSRII II flow cytometer (BD Biosciences) and analyzed by FlowJo software (Tree Star, Inc). Cytokine responses to the two RSV F sub-pools were mock-subtracted respectively and added up to represent cytokine responses specific to RSV F protein. Statistical comparison between groups was done by unpaired *t*-test with Welch’s correction using GraphPad Prism® 8 software.

### Competition ELISAs

Serum samples were analyzed in a competitive binding immunoassay of prefusion or postfusion F protein to D25 antibody, palivizumab, or 4D7 antibody using an AlphaLISA format (PerkinElmer). The following combinations of antibody and F protein were screened: D25/prefusion F protein, palivizumab/postfusion F protein, and 4D7/postfusion F protein. Using a 384-well plate, serum samples were 3-fold serially diluted in a 10-point titration in HiBlock buffer and 10 µL of diluted samples were mixed with 5 µL of AlphaLISA acceptor beads conjugated to prefusion or postfusion F protein (100 µg/mL) in HiBlock buffer. After 30 min incubation at room temperature, 10 µL biotinylated palivizumab (150 pM), D25 (150 pM), or 4D7 (500 pM) antibody diluted in HiBlock buffer was added to each well. After 60 min incubation, 25 µL streptavidin-coated donor AlphaLISA beads was added at 20 µg/mL in HiBlock buffer. All following steps were carried out under subdued lighting. After 90 min incubation at room temperature, the AlphaLISA signal was read on the PHERAStar FS (BMG Labtech) plate reader. Inhibitory antibody titers (IT_50_) were determined by converting raw AlphaLISA counts to percent competition based on the signal relative to 0 and 100% competition wells on control wells. The resulting competition values were fit with a four-parameter fit (bottom fixed to 0 and top fixed to 100) in Graph Pad Prism 7.02. Samples where no dilution hit 50% competition or greater were given the IT_50_ value of 10.

### RSV plaque assay

Lung and nose samples were determined via plaque assay on HEp-2 cells. Briefly, samples were diluted and added in duplicate to 24-well or to 96-well plates as described.^56,57^ For studies conducted using the traditional 24-well plaque assay, HEp-2 cells were pre-seeded and incubated at 37 °C on the day before the assay. On the assay day, virus was added to confluent cell monolayersfor 1 h at 37 °C, the liquid was aspirated and 0.75% methylcellulose was added. Following 5 days at 37 °C, cells were fixed and stained with crystal violet/glutaraldehyde solution. Viral plaques were counted, and titers were expressed as pfu/g of tissue. We also used the higher throughput 96-well assay described in Wen et al.^57^. In this assay format, HEp-2 cells and virus were added to the wells of a 96-well plate at the same time and were incubated for 3 days. Plaques were visualized by immunostaining using a cocktail of anti-F and anti-N antibodies. Primary antibodies were incubated with the fixed cells for 1 h before anti-mouse IgG Alex488 conjugated secondary antibodies (Invitrogen) were added. Viral plaques were then imaged and counted using an EnSight imager reader 2.02 (PerkinElmer). Virus titers were expressed as pfu/g of tissue. Both assay methods were tested on the same set of samples from a cotton rat challenge study and yielded comparable titers.

### Cotton rat histopathology

Cotton rat histopathology was conducted by a blinded pathologist at Sigmovir Biosystems (Rockville, Md). The right lobe of the lung from each cotton rat was dissected and inflated with 10% neutral buffered formalin to their normal volume, and then immersed in the same fixative solution. Following fixation, the lungs were embedded in paraffin, sectioned and stained with hematoxylin and eosin (H&E). The blinded pathologist evaluated the H&E stained slides for evidence of peribronchiolitis (inflammatory cell infiltration around the bronchioles), perivasculitis (inflammatory cell infiltration around the small blood vessels), interstitial pneumonia (inflammatory cell infiltration and thickening of alveolar walls), and alveolitis (cells within the alveolar spaces). Slides were scored on a 0–4 severity scale.

### Cotton rat cytokine expression

mRNA was isolated from the lingular lobe of the lung of each cotton rat. Total RNA was extracted from homogenized lung tissue using the RNeasy purification kit (QIAGEN). One μg of total RNA is used to prepare cDNA using QuantiTect Reverse Transcription Kit (Qiagen). Real-time PCR reactions were conducted to measure the relative level of cytokine mRNAs using the QuantiFast SYBR Green PCR Kit (Qiagen), with final primer concentrations of 0.5 μM. Reactions were performed on a Bio-Rad iCycler for 1 cycle of 95 °C for 3 min, followed by 40 cycles of 95 °C for 10 s, 60 °C for 10 s, and 72 °C for 15 s. The baseline cycles and cycle threshold (Ct) were calculated using the iQ5 software in the PCR Base Line Subtracted Curve Fit mode. Relative quantification of DNA was applied to all samples and the relative expression units were then normalized to the level of β-actin mRNA expressed in the corresponding sample.

### Reporting summary

Further information on research design is available in the Nature Research Reporting Summary linked to this article.

## Supplementary information

Supplemental Material

Reporting Summary

## Data Availability

Merck Sharp & Dohme Corp. a subsidiary of Merck & Co., Inc., Kenilworth, NJ, USA’s data sharing policy, including restrictions, is available at http://engagezone.msd.com/ds_documentation.php through the EngageZone site or via email to dataaccess@merck. Availability of materials: The mRNA vaccines are not publicly available. Samples will not be made available upon request at this time. Readers may contact the corresponding authors to request reagents or materials via a Material Transfer Agreement (MTA), which will be reviewed on a case-by-case basis.
