# Supplementary material for: Modified mRNA/lipid nanoparticle-based vaccines expressing respiratory syncytial virus F protein variants are immunogenic and protective in rodent models of RSV infection
Source: NPJ Vaccines. 2020 Feb 14;5:16. doi: 10.1038/s41541-020-0163-z (PMC7021756; doi:10.1038/s41541-020-0163-z)
Supplement: Supplementary file 1 — Supplemental Material [file 41541_2020_163_MOESM1_ESM.pdf]

Supplementary Files

Supplementary Figure 1. Flow Cytometry of RSV F constructs containing an intact transmembrane domain expressed in Expi293F cells. (a) Gating strategy. Binding to the 4D7 and D25 mAbs (b) or 4D7 and AM14 mAbs (c) is shown for cells transfected with increasing levels of mRNA.

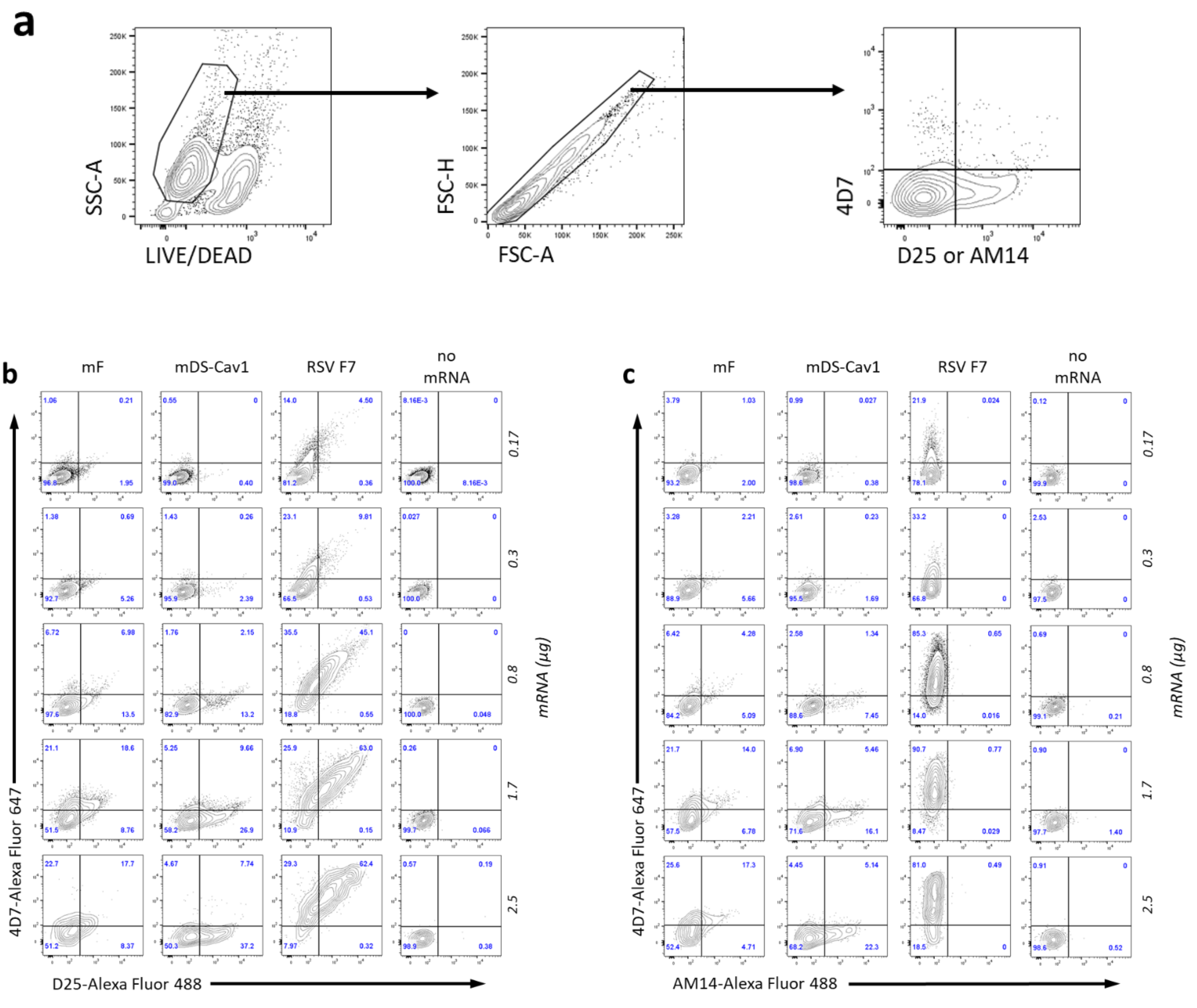

Expi293F cells (ThermoFisher) were transfected with mRNA constructs using the ExpiFectamine 293 Tranfection Kit (ThermoFisher) protocol. Briefly, 80 µL ExpiFectamine 293 Reagent was incubated with 1.5 mL Opti-MEM I Reduced Serum Medium for 5 min at room temperature before mixing with 10 µg of mRNA in 1.5 mL Opti-MEM I Reduced Serum Medium. After 20 min incubation at room temperature, increasing amounts of this mRNA/ExpiFectamine mix (50 µL, 100 µL, 250 µL, 500 µL) were added to Expi293F cells incubated in 24-well plates (0.6 ml/well, 1.8 x 10^6 cells/well) in Expi293 Expression Medium. Cells were placed on an orbital shaker (125 rpm) in a cell incubator at 37°C for 24 h. At that time, cell supernatants were collected and kept at -20°C until ELISA analysis and cells were stained for FACS analysis.

After transfection, Expi293 cells (0.5 x 10^6 cells) were pelleted in a V-bottom 96 well plate and resuspended in PBS (250 µL). Live/Dead Fixable Aqua Dead Cell stain (ThermoFisher) reconstituted in DMSO per manufacturer's instructions was added to each well (1 µL /well) and incubated at room temperature in the dark for 30 min. Cells were pelleted, washed twice with Stain Buffer (FBS) (300 µL/well, BD Biosciences), and added to a V-bottom 96 well plate (0.5 x 10^6 cells/well). Cells were resuspended in Stain Buffer (FBS) with D25-Alexa Fluor 488 or AM14-Alexa Fluor 488 and 4D7-Alexa Fluor 647, or isotype control antibodies (each at 5 µg/mL) and incubated for 15 min on ice in the dark. Cells were then pelleted and washed twice with Stain Buffer (FBS) (300 µL/well), resuspended in Stain Buffer (FBS) (200 µL/well) and analyzed in a BD LSR II flow cytometer.

The plots of cells transfected with four different amounts of mRNA (0.17, 0.3, 0.8, and 1.7 mg) are shown. (a) Live singlet cells were identified to analyze D25 and 4D7 or AM14 and 4D7 staining levels. (b) Binding to 4D7 is shown on the Y-axis, or upper half of each plot, while binding to D25 is shown in the X-axis or right-hand side of each plot. Cells expressing proteins that bind to both 4D7 and D25 are identified in the upper right-hand quadrant of each plot. By qualitative analysis, mF and RSV F7-

transfected cells showed the highest level of binding to 4D7 and each had a population of cells that bound both D25 and 4D7. Cells transfected with mDS-Cav1 bound predominantly D25 with little 4D7 binding. (c) Binding to 4D7 is shown on the Y-axis, while binding to AM14 is shown on the X-axis. Cells expressing proteins that bind to both 4D7 and AM14 are identified in the upper right-hand quadrant of each plot. mDS-Cav1 had the highest fraction of cells binding AM14 and not 4D7 (22.3% of cells at 2.5 mg), with a somewhat higher fraction of cells binding D25 and not 4D7 (37% of cells at 2.5 mg), suggesting the translated protein was primarily in the prefusion conformation and largely trimeric. mF appeared to include a mixture of conformations including cells binding 4D7, D25, and to a lesser extent, AM14. RSV F7 mRNA was translated into a protein with strong 4D7 and D25 binding with little to no AM14 binding, possibly indicating a monomeric, prefusogenic conformation.

Supplementary Figure 2. Bio-Layer Interferometry (BLI) Analysis of DS-Cav1 protein (A) in comparison with sF protein (B)

A. B.

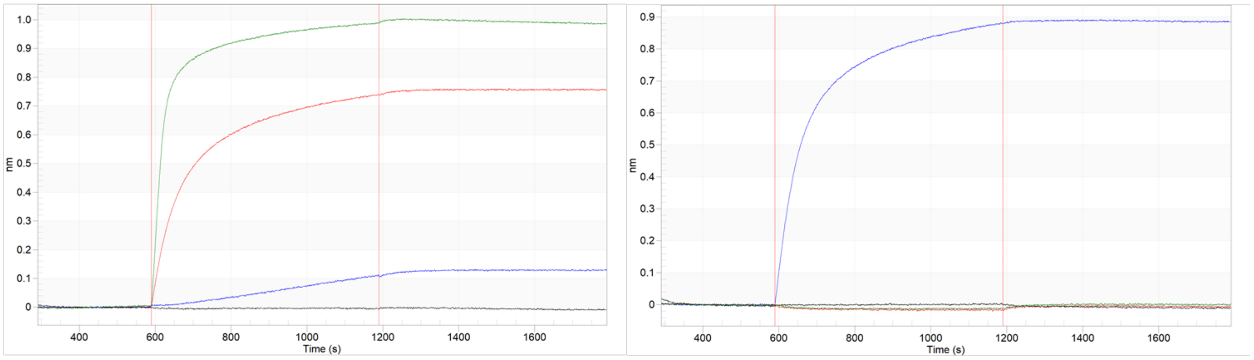

Protein	Antibody Ligand	Curve Color
sDS-Cav1	AM14	green
sDS-Cav1	D25	red
sDS-Cav1	4D7	blue
non-RSV soluble protein	AM14 (representative of all antibody ligands)	black

Protein	Antibody Ligand	Curve Color
sF	AM14	green
sF	D25	red
sF	4D7	blue
non-RSV soluble protein	AM14 (representative of all antibody ligands)	black

A Pall ForteBio Octet Red96e instrument was used to confirm the conformation of sDS-Cav1 protein by assessing the association between RSV F variants, sDS-Cav1 (A) and sF (B), and antibodies against various RSV F conformations. A non-RSV soluble protein was included as a negative control. All assays were completed with agitation at 1000 rpm. Assays were performed at 25°C in flat bottom black 96-well plates (Greiner Bio-One) with evaporation covers. All antibodies and proteins were diluted in 1X Kinetics Buffer (1X KB: 10X Kinetics Buffer (Pall ForteBio) diluted 1:10 in phosphate-buffered saline (PBS)). The final volume for all solutions in the plate was 200 µl/well. Anti-human IgG Fc Capture sensors (AHC: Pall ForteBio) were stabilized with 5 sec alternating pulses of 10 mM glycine pH 1.75 and 1X KB for 3 cycles, baselined for 60 sec in 1X KB and then loaded with antibodies at a concentration of 5 ug/ml for 200 sec.

Biosensor tips were equilibrated for 300 sec in 1X KB before measurement of association with RSV F and non-RSV soluble proteins (25 nM) for 600 sec. Proteins were allowed to dissociate for 600 sec. Data analysis and curve generation were completed using ForteBio Data Analysis 10.0 software. To account for systemic baseline drift, all data were background subtracted with the measurement of a reference well, an antibody-loaded sensor incubated in 1X KB buffer alone. All processed data was y-axis aligned to baseline. In (A), sDS-Cav1 protein is shown to bind to the prefusion-indicating D25 and AM14 mAbs with low levels of binding to the 4D7 mAb; in contrast in (B) the non-prefusion stabilized sF protein binds strongly to the 4D7 mAb but does not bind D25 or AM14.

Supplementary Figure 3: Representative example and gating strategy for Intracellular Cytokine Staining Data.

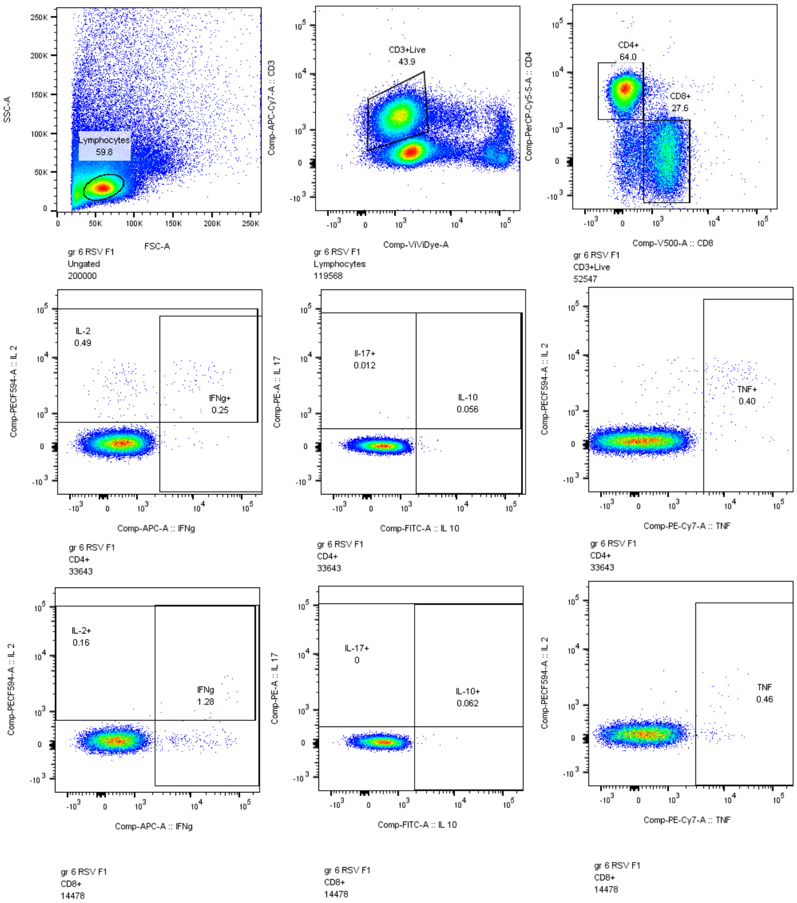

Lymphocytes were gated based on size and granularity, followed by a gate of CD3+ viable cells. Next, gates were placed on CD4 or CD8 T cells. For each subset (CD4 row 2, or CD8 row 3), the cells were plotted for cytokine expression with a gate on the single positives for each (IFNγ, IL-2, IL-17, IL-10, TNF). This gating strategy applies to the data in Figure 3.

Supplementary Figure 4: qPCR measurement of RSV RNA in Cotton Rat Nose (a) and Lung (b)

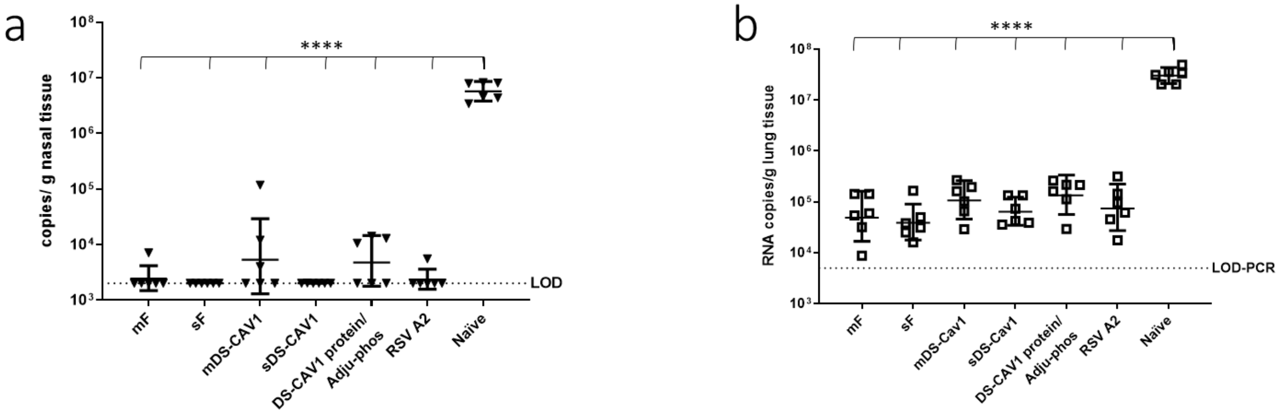

In the cotton rat experiment described in Figure 4, RNA was harvested from nose and lung four days following challenge. Lung and nose were harvested from cotton rats four days following challenge and homogenized in 3ml of HBSS containing 10% SPG. The homogenates were then centrifuged for clarification and frozen until further use. RNA was extracted from the homogenates using the Maxwell® 16 Viral Total Nucleic Acid Purification Kit (Promega) and the AS2000 Maxwell® 16 Instruments according to the manufacturer's instructions. RSV mRNA was then quantified by RT-qPCR using the Quantitect® Probe Rt-PCR kit (1000) (Qiagen). Primers and probes targeted the RSV N gene and were 5' CTC ATT TTC CTC ACT TCT CCA GTG T 3' (F), 5' CTT GAT TCC TCG GTG TAC CTC TGT 3' (R), 5'FAM-TCC CAT TAT GCC TAG GCC AGC AGC A (BHQ1) (probe). Final primer concentration was 300nM and final probe concentration was 200nM. Reactions were performed on a Stratagene Mx3005P thermocycler for 30 min of reverse transcription at 50C, followed by 1 cycle of 95ºC for 15 min, followed by 40 cycles of 94ºC for 15 sec, and 62ºC for 60 sec. The baseline cycles and cycle threshold (Ct) were calculated using Stratagene Mx3005p software. Copy number was determined based on an RSV A standard generated from purified RSV A using the QIAGEN OneStep RT-PCR kit and the RNA copy number per gram of tissue is plotted for each animal (N=6 per group). The geometric mean and 95% confidence intervals are shown, and the dotted line on each graph indicates the limit of detection for the assay. All vaccinated

groups had significantly reduced RSV RNA copies in both nose and lung when compared with the unvaccinated, challenged animals ($p<0.0001$ by two-sided unpaired t-test conducted using GraphPad Prism soaftware). RSV RNA copy number in the nose and lung of mRNA/LNP or DS-Cav1 protein vaccinated animals was comparable to animals in the RSV A2 pre-treated group.

Supplementary Figure 5a: Neutralizing Antibody Titers from Cotton Rat ERD Histopathology Study

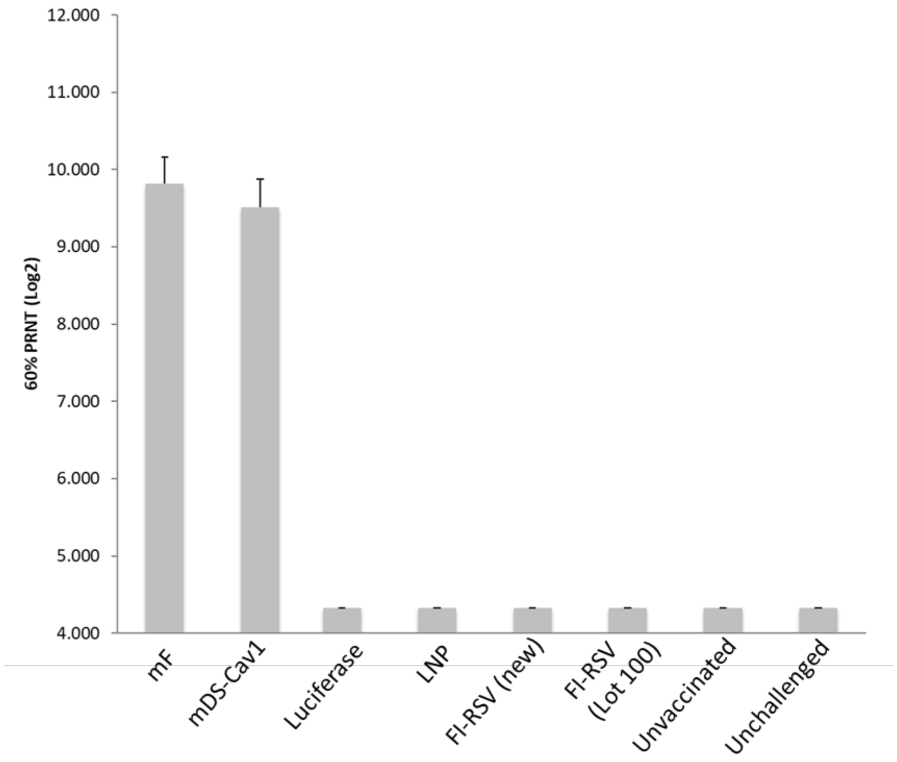

Serological assays were conducted in the cotton rat ERD histopathology study described in Figure 7. Neutralizing Antibody titers from sera taken 4 weeks following the second dose is shown. Titers were calculated at Sigmovir, Inc. The neutralizing antibody titers were determined by plaque reduction assay using RSV-A2 and HEp-2 cells. Serum samples were incubated with 25-50 pfu of RSV/A2 for 1 hour at room temperature and inoculated in duplicate onto confluent HEp-2 monolayers in 24 well plates. After one hour incubation at 37°C in a 5% CO_2 incubator, the wells were overlayed with 0.75% methylcellulose medium. After 4 days, the overlays were removed and the cells were fixed and stained with 0.1% crystal violet. Neutralizing antibody titers were determined at the 60% reduction end-point of the virus control using the statistics program "plqrd.manual.entry". The geometric means ± standard error for all animals in a group are shown (N=10).

Supplementary Figure 5: Protection in Cotton Rat ERD histopathology study in lung (b) and nose (c)

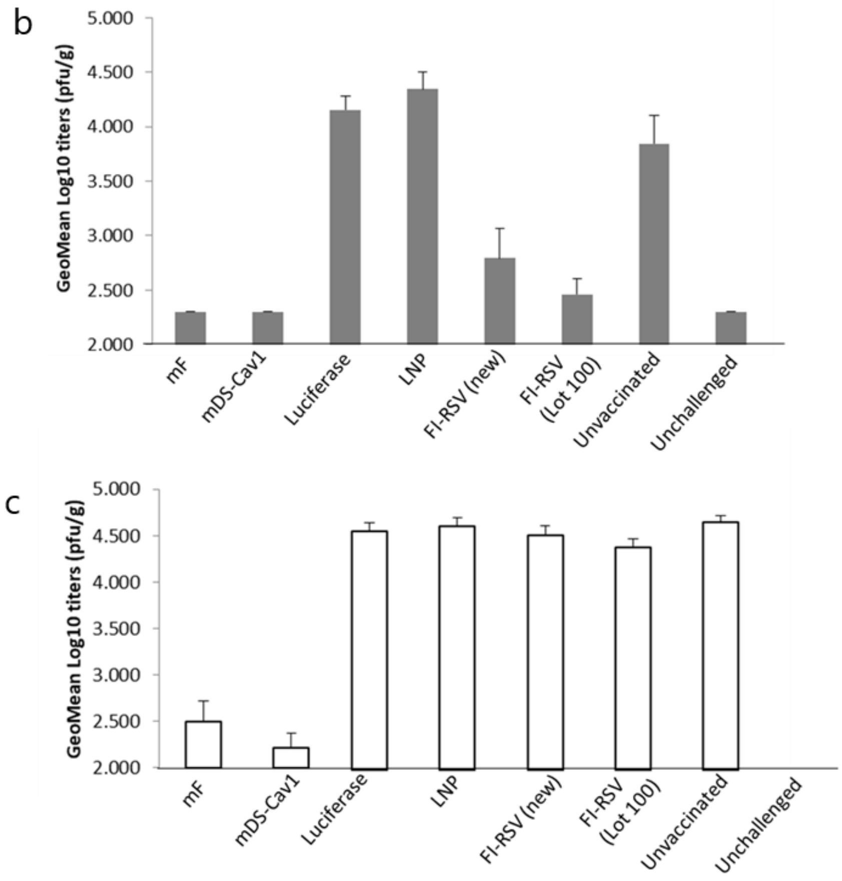

RSV titers were measured in the cotton rat ERD histopathology study described in Figure 7. Cotton rat titers in the lung (b) and nose (c) of each animal (N=10) were measured four days following RSV-A2 challenge at Sigmovir, Inc. To measure RSV titer, lung and nose homogenates were clarified by centrifugation and diluted in EMEM. Confluent HEp-2 monolayers were infected with diluted homogenates in 24 well plates (N=2 for each sample). After one hour incubation at 37°C in a 5% CO_2 incubator, the wells were overlayed with 0.75% methylcellulose medium, incubated for 4 days, and stained with 0.1% crystal violet as described above. Plaques were counted manually and viral titers were determined for each sample. Geometric mean titers are presented in log10 pfu/gram of tissue (+/- SEM).

Supplementary Table 1 Raw histopathology data from Cotton Rat ERD Histopathology Study

Group	*Peribronchiolitis*	*Perivasculitis*	*Interstitial Pneumonia*	*Alveolitis*
mF	2	1	0	1
	1	1	0	0
	2	1	0	1
	2	1	0	1
	2	2	2	2
	1	1	1	1
	2	2	0	1
	2	2	1	1
	2	1	0	0
	2	1	1	1
mDS-Cav1	2	2	1	1
	2	1	0	1
	2	1	0	0
	2	2	1	1
	2	2	1	2
	2	1	1	2
	2	1	1	1
	2	1	1	1
	2	2	1	1
	2	2	0	1
Luciferase	2	1	0	0
	2	2	0	0
	2	2	1	2

	2	2	0	0
	2	2	1	1
	2	2	0	1
	2	2	2	2
	3	2	2	2
	2	2	0	1
	2	2	1	1
LNP	3	1	0	0
	2	2	0	1
	3	2	1	1
	3	2	0	1
	2	2	1	2
	2	2	0	1
	2	1	0	1
	2	1	0	0
	3	2	1	1
	3	2	0	1
FI-RSV (new)	3	2	1	2
	3	2	2	2
	2	2	2	2
	3	2	2	2
	3	2	1	1
	3	2	1	2
	3	2	2	2
	3	3	2	3
	3	2	2	2
	3	1	0	1

FI-RSV (Lot 100)	3	3	2	3
	2	2	0	1
	3	3	1	1
	3	2	1	2
	3	2	2	3
	3	2	0	1
	3	2	1	2
	3	2	2	3
	3	2	1	1
	3	2	0	1
Unvaccinated	2	2	0	1
	2	2	1	1
	2	2	1	1
	2	2	2	2
	2	2	2	2
	2	1	0	1
	2	1	0	0
	3	2	1	1
	2	2	1	1
	2	2	0	0
Unchallenged	1	1	0	0
	1	1	0	1
	2	1	1	1
	2	1	0	1
	1	1	0	0
	1	1	0	0
	2	1	0	0

	1	2	0	1
	1	0	0	0
	1	0	0	0
